# Vulvovaginal yeast infections during pregnancy and perinatal outcomes: systematic review and meta-analysis

**DOI:** 10.1186/s12905-023-02258-7

**Published:** 2023-03-21

**Authors:** Ranjana M. S. Gigi, Diana Buitrago-Garcia, Katayoun Taghavi, Cara-Mia Dunaiski, Janneke H. H. M. van de Wijgert, Remco P. H. Peters, Nicola Low

**Affiliations:** 1 Institute of Social and Preventive Medicine, University of Bern, Bern, Switzerland; 2 Research Unit, Foundation for Professional Development, East London, South Africa; 3 Graduate School for Cellular and Biomedical Sciences, University of Bern, Bern, Switzerland; 4 Graduate School for Health Sciences, University of Bern, Bern, Switzerland; 5 School of Health Sciences, Namibia University of Sciences and Technology, Windhoek, Namibia; 6 Julius Center for Health Sciences and Primary Care, University Medical Center Utrecht, Utrecht University, Utrecht, Netherlands; 7 Department of Medical Microbiology, University of Pretoria, Pretoria, South Africa; 8 Division of Medical Microbiology, University of Cape Town, Cape Town, South Africa

**Keywords:** Vaginal candida, Vaginal yeast, Pregnancy, Preterm birth, Adverse perinatal outcomes, Systematic review

## Abstract

**Background:**

Vulvovaginal yeast infections in pregnancy are common and can cause extensive inflammation, which could contribute to adverse pregnancy outcomes. Symptomatic yeast infections are likely to cause more inflammation than asymptomatic. The objective of this study was to investigate associations between symptomatic and asymptomatic vulvovaginal yeast infections in pregnancy and perinatal outcomes.

**Methods:**

We did a systematic review and searched eight databases until 01 July 2022. We included studies reporting on pregnant women with and without laboratory confirmed vulvovaginal yeast infection and preterm birth or eight other perinatal outcomes. We used random effects meta-analysis to calculate summary odds ratios (OR), 95% confidence intervals (CI) and prediction intervals for the association between yeast infection and outcomes. We described findings from studies with multivariable analyses. We assessed the risk of bias using published tools.

**Results:**

We screened 3909 references and included 57 studies. Only 22/57 studies reported information about participant vulvovaginal symptoms. Preterm birth was an outcome in 35/57 studies (49,161 women). In 32/35 studies with available data, the summary OR from univariable analyses was 1.01 (95% CI 0.84–1.21,* I*^2^ 60%, prediction interval 0.45–2.23). In analyses stratified by symptom status, we found ORs of 1.44 (95% CI 0.92–2.26) in two studies with ≥ 50% symptomatic participants, 0.84 (95% CI 0.45–1.58) in seven studies with < 50% symptomatic participants, and 1.12 (95% CI 0.94–1.35) in four studies with asymptomatic participants. In three studies with multivariable analysis, adjusted ORs were greater than one but CIs were compatible with there being no association. We did not find associations between vulvovaginal yeast infection and any secondary outcome. Most studies were at high risk of bias in at least one domain and only three studies controlled for confounding.

**Conclusions:**

We did not find strong statistical evidence of an increased risk for preterm birth or eight other adverse perinatal outcomes, in pregnant women with either symptomatic or asymptomatic vulvovaginal yeast infection. The available evidence is insufficient to make recommendations about testing and treatment of vulvovaginal yeast infection in pregnancy. Future studies should assess vulvovaginal symptoms, yeast organism loads, concomitant vaginal or cervical infections, and microbiota using state-of-the-art diagnostics.

**Systematic review registration:**

PROSPERO CRD42020197564

**Supplementary Information:**

The online version contains supplementary material available at 10.1186/s12905-023-02258-7.

## Background

Vulvovaginal yeast infections in pregnancy are common and can cause extensive inflammation, which could contribute to adverse perinatal outcomes [1]. Preterm birth is the most common cause of neonatal death worldwide [2]. The causes of preterm birth include socio-economic factors, underlying maternal conditions, foetal conditions, and infectious causes [3]. Infectious causes include upper, and possibly lower, genital tract infections [4], with some evidence that early preterm birth is more commonly infection-related than late preterm birth [5]. Vulvovaginal yeast infections caused by *Candida* species, are more common in pregnant women than non-pregnant women [1, 6, 7], potentially because of hormonal and immunological changes that occur during pregnancy [8]. It is not known whether yeast organism loads are higher in pregnant than non-pregnant women or whether they are associated with levels of inflammation or adverse perinatal outcomes [9].

Microorganisms in the female genital tract may have direct pathogenic effects in pregnancy through infection of the amniotic cavity and/or through stimulating inflammatory cascades [4]. Besides prostaglandins, chemokines and pro-inflammatory cytokines can ripen the cervix and induce contractions [10]. These pathways may be activated by infections during pregnancy and lead to preterm birth [10]. Yeast infections in the female genital tract cause inflammation and therefore increase proinflammatory mediators in the vaginal fluid, such as interleukin-8, which have been associated with preterm birth [11, 12]. A systematic review reporting on studies of asymptomatic *Candida* colonization published up to May 2020 did not find an association with adverse pregnancy outcomes [13]. Symptomatic yeast infections are likely to cause more inflammation than asymptomatic infection, however. The objective of this study was to investigate associations between both symptomatic and asymptomatic vulvovaginal yeast infections in pregnancy and preterm birth and other perinatal outcomes.

## Methods

We did a systematic review and registered the protocol in the International Prospective Register of Systematic Reviews (PROSPERO: CRD42020197564). We followed the Preferred Reporting Items for Systematic reviews and Meta-Analyses (PRISMA) 2020 guidelines for reporting the review [14].

### Search strategy

We searched Medline (Ovid), PubMed, Embase (Ovid), the Cochrane Library, CINAHL, African Index Medicus, LILACS and ClinicalTrials.gov (Supplementary search strategy, Additional File 1) from inception until 01 July 2022 without language restrictions. Additional studies were retrieved by checking reference lists of relevant articles. For articles in languages not spoken by our team, we used DeepL to translate [15].

### Eligibility criteria

We included cross-sectional, case-control, cohort studies and clinical trials. Eligible studies were those that included pregnant women who had a laboratory test for vulvovaginal yeast infection before the outcome occurred and reported on pre-defined outcomes. We excluded studies in which all included pregnant women had confirmed vulvovaginal yeast infection without a control group for comparison.

### Study selection

One reviewer (RG) screened all titles and abstracts, and a second reviewer (DB) cross-checked a random sample of 10%. Discrepancies were resolved by discussion. The full texts of potentially eligible manuscripts were assessed by one reviewer (RG) and all were verified by another (DB, KT, CD, NL). Final decisions were made by consensus or adjudication by a third reviewer (NL).

### Outcome definitions

Preterm birth, defined as birth before 37 completed weeks of pregnancy, was our primary outcome [16]. Secondary outcomes included spontaneous abortion (delivery of a dead foetus before 22 completed weeks of pregnancy), stillbirth (delivery of a dead foetus after 22 completed weeks of pregnancy), preterm premature rupture of membranes (spontaneous tearing of the membranes surrounding the foetus before 37 weeks of gestation), premature rupture of membranes (spontaneous tearing of the membranes surrounding the foetus any time before the onset of obstetric labour), low birth weight (less than 2500 g), small for gestational age (birth weight below the 10th centile for gestational age), inflammation of the placenta or uterus (endometritis, chorioamnionitis, villitis, or funisitis), and neonatal death (death of a live-born infant during the first 28 completed days of life) [3, 16, 17].

### Data extraction

We designed online forms in the Research Electronic Data Capture software (REDCap) [18] to extract data about study design, basic study population characteristics, symptom status, risk factors, laboratory characteristics, and study outcomes (Supplementary REDCap data extraction forms, Additional File 2). Symptom status was defined as the presence or absence of vulvovaginitis, curdy white discharge, vulval or vaginal itch or vaginal discharge, either self-reported or clinician-observed. One reviewer (RG, DB, KT, CD, or NL) extracted data into the forms and a second reviewer (RG, DB, KT, CD, or NL) verified all the extracted data, with discrepancies resolved by discussion. We contacted the authors for data that were not reported in the article. If we did not receive a reply by 01 November 2022, we excluded the study from the review. If vulvovaginal specimens were taken at multiple timepoints during pregnancy, we extracted the data from the first timepoint as this included the largest number of participants and was most consistent with the timepoint of other included studies. If more than one article reported on the same study population, we considered these as a single study, but extracted relevant information from any linked publication.

### Risk of bias assessment

Two reviewers (RG, DB, KT, CD, or NL) assessed the risk of bias independently using published tools [19, 20], developed by the Clarity group of evidence-based healthcare experts (https://www.clarityresearch.ca). For clinical trials and cross-sectional studies, we assessed the risk of bias with the tool designed for cohort studies, which included the relevant questions. We added two relevant questions: (1) was the study designed specifically to assess the association between vulvovaginal yeast infection and pregnancy outcomes, and (2) did the authors control for confounding in their analysis. Where there were ten or more studies, we generated funnel plots to assess evidence for publication bias and other small study biases [21].

### Data synthesis

We assessed heterogeneity between studies visually with forest plots and used the *I*^*2*^ statistic to describe the proportion of variability other than that due to chance [22]. Where appropriate, we conducted random effects meta-analysis to calculate summary odds ratios (OR) and 95% confidence intervals (CI), which shows the average size of the association between yeast infection and each outcome in included studies. Where there were three or more studies, we also calculated the 95% prediction interval, which gives a range for the strength of association in a future study [22]. Meta-analyses and forest plots were produced using R 4.1.2 [23]. The completeness and comprehensiveness of symptom reporting varied between studies, therefore we stratified studies reporting on preterm birth into five groups. Few studies reported the proportion of women with symptoms and one enrolled only symptomatic participants [24]. We split these studies at the halfway mark. The categories were: (1) no symptoms (observed or self-reported), (2) < 50% participants with symptoms, (3) ≥ 50% participants with symptoms, (4) participants with symptoms included but proportion unknown, and (5) symptom status not reported. We also stratified studies reporting about preterm birth by study design, income setting (according to The World Bank classification) [25], and human immunodeficiency virus (HIV) infection status. Post-hoc, we stratified studies by trimester of testing and diagnostic methods used. For studies in which authors conducted a multivariable analysis, we plotted the unadjusted and adjusted OR (and 95% CI) and recorded the variables that were adjusted for in each study.

## Results

**Fig. 1:**
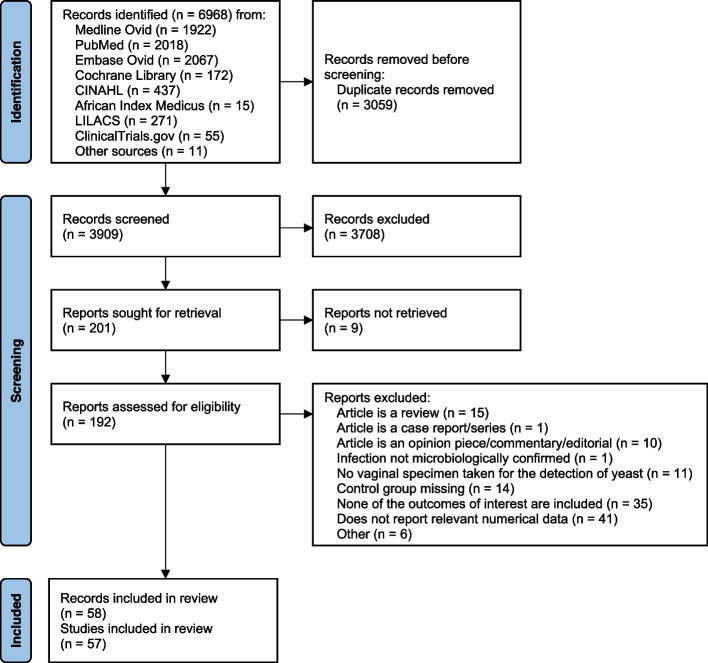
Flowchart of the selection process of articles

We screened 3909 references and included 57 different studies. The main reasons for exclusion were due to studies not reporting any of our outcomes of interest, or having insufficient numerical data (Fig. 1). Table 1 summarises characteristics of the included studies, from 27 different countries, published between 1969 and 2022 with a median sample size of 347 participants (interquartile range, IQR 200–1000). Five were cross-sectional studies [24, 26–29], 15 were case–control studies [30–44], 35 were cohort studies [45–81], and two were randomised clinical trials [82, 83] (Table 1).

**Table 1:** Summary of characteristics of studies included in the systematic review

Participant characteristics	Total	No symptoms	Asymptomatic and symptomatic	Only symptomatic	Not reported
Number of studies, n	57	5	16	1	35
Study design, n					
Cohort	35	3	12	0	20
Case-control	15	0	3	0	12
Cross-sectional	5	0	1	1	3
Clinical trial	2	2	0	0	0
Publication, year median (IQR)	2012 (1995–2018)	2014 (2011–2015)	2016 (1997–2019)	2020	2008 (1995–2016)
Number of women, total (median, IQR)	71,500 (374, 200–1000)	13,923 (500, 347–4429)	22,574 (331, 202–1220)	258	34,745 (300, 156–912)
Outcomes, reported, n					
Preterm birth	35	4	10	1	20
Spontaneous abortion	5	1	2	0	2
Stillbirth	4	1	3	0	0
PPROM	11	0	3	0	8
PROM	15	1	5	0	9
Low birth weight	11	1	5	0	5
Small for gestational age	1	0	0	0	1
Inflammation of the placenta or uterus	3	0	1	0	2
Neonatal death	1	0	1	0	0
Time of testing, n					
1^st^ trimester	12	2	2	0	8
2^nd^ trimester	24	3	4	0	17
3^rd^ trimester	27	2	5	1	19
not reported	18	1	8	0	9
Specimen type, n					
Vaginal swab	41	4	11	1	25
Endocervical swab	14	0	2	0	12
Other^a^	5	1	3	0	1
not reported	4	0	1	0	3
Diagnostic method, n					
Microscopy	30	2	12	0	16
Culture	34	3	10	1	20
PCR	3	1	1	0	1
not reported	7	0	0	0	7

*IQR* Interquartile range, *PCR* Polymerase chain reaction, *PPROM* Preterm premature rupture of membranes, *PROM* Premature rupture of membranes

^a^Vaginal smears, endocervical smears, vaginal wash, cervicovaginal fluid

The total number of studies included is 57. The totals for each item can sum to more than 57 because a study might have reported on more than one item

We included 58 records that reported on 57 different studies (Table 1). Among the 57 included studies, 22 (38.6%) described whether participants had reported symptoms and assessed symptom status. Of these 22 studies, 17 included participants with symptoms (14 reported the percentage of participants with symptoms) [24, 29, 39, 42, 43, 45–47, 58, 59, 65, 67, 69, 77–79, 84] and five studies included only participants without symptoms [55–57, 70, 82, 83]. Reporting about symptom in terms of self-reported or clinician-observed symptoms and the level of detail varied between studies. Of the 22 studies, eight studies reported symptoms observed by the clinician [42, 45, 46, 54, 59, 67, 69, 77], two only self-reported [43, 78], five studies both [29, 58, 65, 79, 82] and seven did not reported if the symptoms were self-reported or clinician-observed [24, 39, 47, 55–57, 70, 83]. Of the 17 studies which included participants with symptoms, six studies report proportions of different types of vaginal discharge and other symptoms which are associated with genital tract infections [29, 43, 47, 54, 65, 67], ten reported if symptoms from a defined group of symptoms were present or absent [24, 39, 42, 45, 58, 59, 69, 77–79], one did not report the type or definition of symptoms [46]. Where symptom status was reported, the information was captured at baseline. The main laboratory methods used were culture and microscopy (Table 1). Thirty-one studies reported on the yeast species detected, and *Candida albicans* was most frequently reported. Of the 57 studies, 39 provided information about the timing of testing during pregnancy (Table 1). Only five studies tested at multiple timepoints during pregnancy for all or a subset of participants [55, 56, 58, 60, 63, 83]. Other infections are reported in 46 studies but only nine report outcome data on co-infections of vulvovaginal yeast infections [29, 37, 39, 55, 56, 59, 70, 74, 78, 82].

**Fig. 2:**
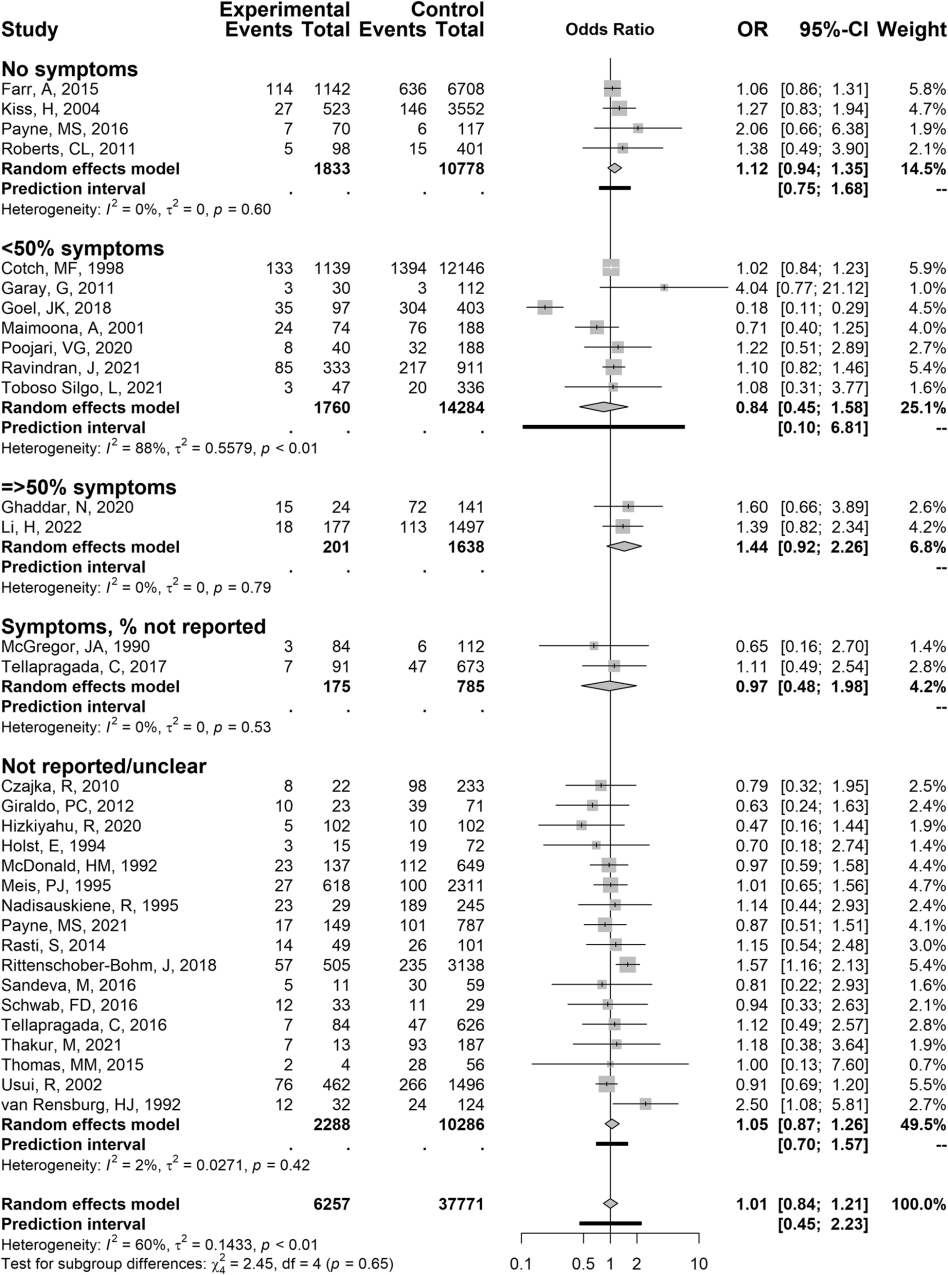
Forest plot of meta-analysis vulvovaginal yeast infections and preterm birth stratified by symptom status. Legend: vertical line, line of no association (odds ratio 1.0); horizontal line, 95% confidence interval; vertical line inside the box, point estimate of odds ratio; grey box, study size; diamond, summary estimate with 95% confidence interval; black bar, 95% prediction interval. To the left of the line of no association, preterm birth was less likely in women with vulvovaginal yeast infection; to the right of the line of no association, preterm birth was more likely

**Fig. 3:**
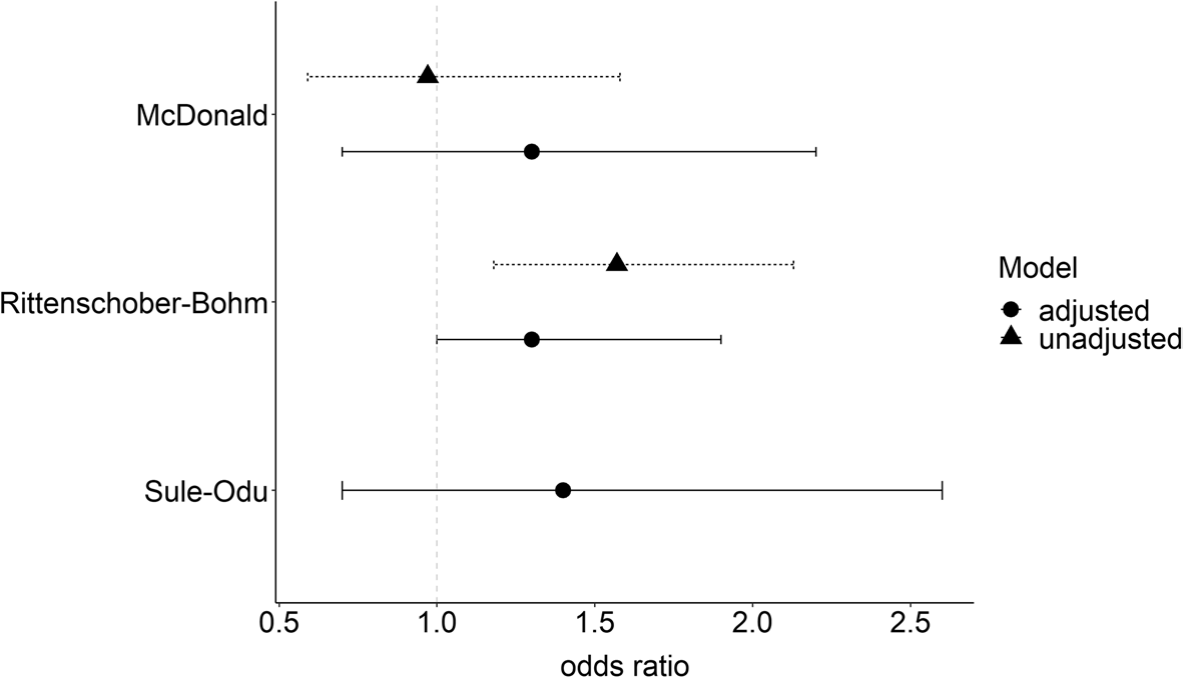
Forest plot of unadjusted and adjusted estimates on vaginal yeast infections and preterm birth. Legend: horizontal lines, 95% confidence intervals; dot/triangle, odds ratio. Unadjusted estimates were calculated from raw numbers (not provided for Sule-Odu [81]); adjusted estimates are those reported by the authors. McDonald [61] adjusted for previous preterm delivery (> 20 weeks), previous midtrimester miscarriage, multiple pregnancy, cervical incompetence, polyhydramnios, uterine malformation, pyelonephritis during pregnancy, *Gardnerella vaginalis*, *Ureaplasma urealyticum*, *Mycoplasma hominis*, *Bacteroides* spp, *Peptostreptococcus* spp, Group B streptococci, *Escherichia coli*, *Klebsiella* spp, *Staphylococcus aureus*, *Haemophilus* spp, yeast. Rittenschober-Bohm [74] adjusted for bacterial vaginosis, vaginal candidiasis, history of preterm birth, smoking, age, *Ureaplasma urealyticum* positive, *U. parvum* positive, *U. urealyticum* and *U. parvum* positive. Sule-Odu [81] adjusted for age, parity, educational level, socioeconomic status, birth weight, gestational age at delivery

The primary outcome, preterm birth, was reported in 35 studies with data on 49,161 women. Univariable data were available for 32 studies and data from a multivariable model for three studies [61, 74, 81]. Figure 2 shows the studies reporting univariable data according to reported symptom status and Fig. 3 studies with multivariable data. For all included studies reporting on preterm birth, we found a summary OR of 1.01 (95% CI 0.84–1.21,* I*^2^ 60%, prediction interval 0.45–2.23, 32 studies). Within groups according to symptom status, heterogeneity was mostly low (Table 2). The OR for preterm birth was 1.44 (95% CI 0.92–2.26,* I*^2^ 0%) in two studies with ≥ 50% participants with symptoms [24, 29], 0.84 (95% CI 0.45–1.58,* I*^2^ 88%, prediction interval 0.10–6.81) in seven studies with < 50% participants with symptoms [43, 45–47, 65, 78, 79], 1.12 (95% CI 0.94–1.35,* I*^2^ 0%, prediction interval 0.75–1.68) in four studies only with participants without self-reported or observed symptoms [55, 56, 70, 82, 83], and 1.05 (95% CI 0.87–1.26,* I*^2^ 2%, prediction interval 0.70–1.57) in 17 studies in which symptom status was not reported [27, 28, 32, 37, 40, 44, 50, 53, 61, 63, 64, 66, 68, 72, 74–76]. The prediction intervals for all groups with sufficient data included the null value. Of the three studies not included in the meta-analysis, one reported 90% confidence intervals (OR 0.88, 90% 0.61–1.27) [49], one assessed three testing timepoints and analysed the data in five groups, concluding from univariate logistic regression that different ‘trends’ of vulvovaginal candidiasis were not associated with preterm birth [60], and one only reported adjusted estimates [81].

**Table 2:** Association between vulvovaginal yeast infection and secondary outcomes, from univariate analysis

Outcome	Number of studies included in meta-analysis	OR	95% CI	*I*^*2*^ value
Spontaneous abortion [26, 55, 56, 58, 62]	4	1.01	0.60–1.72	44%
Stillbirth [45, 55, 56, 69]	3	1.09	0.66–1.78	0%
Preterm premature rupture of membranes [28, 30, 34, 35, 41, 42, 45, 48, 61, 65]	10	0.82	0.51–1.31	70%
Premature rupture of membranes [29, 31, 33, 36, 38, 39, 41, 45, 46, 51, 57, 66, 71, 79, 80]	15	1.02	0.74–1.41	64%
Low birth weight [29, 45, 52, 54–56, 59, 66, 72, 79]	9	1.02	0.78–1.34	41%
Small for gestational age [49]	1	1.80	90% CI 1.17–2.77	N/A
Inflammation of the placenta or uterus [41, 44]	2	1.20	0.59–2.41	0%
Neonatal death [45]	1	1.08	0.46–2.53	N/A

*OR* Odds ratio, *CI* Confidence interval, *N/A* Not applicable for strata with a single study

Heterogeneity assessed by the *I*^*2*^ test was very low. One study by Goel et al. was an outliner in which vulvovaginal yeast infections were associated with lower odds of preterm birth [47]. Goel et al. (2018) enrolled 500 pregnant women at any gestational age from a medical institute in India. Preterm risk factors were not part of the enrolment criteria. This study reported that 67.8% of women had a preterm delivery, but there was no definition of preterm delivery nor any discussion about the high number of preterm deliveries [47].

There was no strong statistical evidence of an association in meta-analyses about vulvovaginal yeast infections and preterm birth in pre-specified subgroups stratified by study design and income setting or in post-specified subgroups stratified by diagnostic methods used and time of testing (Supplementary forest plots of stratified meta-analyses, Additional File 3). Only four studies reported on the inclusion or exclusion of women living with HIV and results for the association between yeast infection and preterm birth were not stratified according to HIV infection status (three excluded HIV positive women [67, 72, 76], one included HIV positive women but did not stratify results by HIV status [78]).

There were three studies reporting adjusted estimates from multivariable analysis (Fig. 3) [61, 74, 81]. McDonald et al. and Rittenschober-Bohm et al. adjusted for known preterm risk factors and infections and Sule-Odu et al. (who reported only the adjusted OR) adjusted for socioeconomic factors but not for infections. CIs for all adjusted ORs included the null value. The CIs of the adjusted ORs overlapped with those of the unadjusted estimates in the studies by McDonald et al. and Rittenschober-Bohm et al. and the direction of the OR after adjustment for potential confounders was not consistent.

We did not find strong statistical evidence of associations between vulvovaginal yeast infection and any secondary outcome, reported in 1–15 studies (Table 2 and Supplementary forest plots of secondary outcomes, Additional File 4). For one study that reported on the outcome small for gestational age, authors reported a 90% CI (OR 1.80, 90% CI 1.17–2.77) [49].

### Risk of bias

**Fig. 4:**
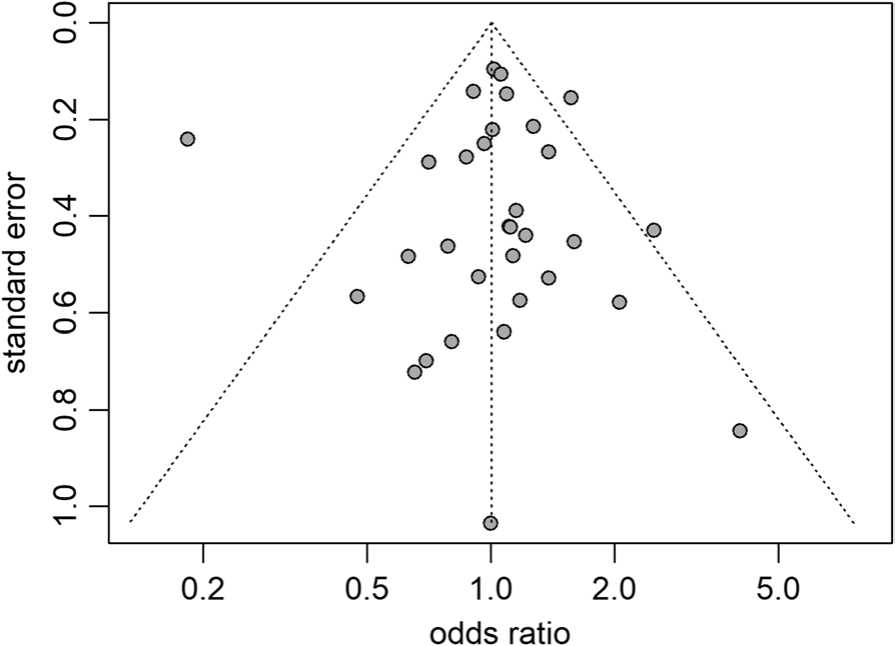
Funnel plot of published studies reporting on preterm birth. Legend: circle, represents one study; triangle, region where 95% of the data points would lie in absence of small study biases; vertical dashed line, odds ratio from meta-analysis

We judged most studies to have a high risk of bias in at least one domain (Supplementary summary of risk of bias assessment, Additional File 5). There were concerns about the participant selection, diagnostic tools used to detect vulvovaginal yeast infections, frequency of testing during pregnancy and incomplete reporting. As described, only three studies included a multivariable analysis to control for confounding. For the three outcomes (preterm birth, preterm premature rupture of membranes, premature rupture of membranes) with more than ten included studies, funnel plots were symmetrical, indicating low risk of small study biases (Fig. 4 and supplementary funnel plots of secondary outcomes, Additional File 6) [21].

## Discussion

### Summary of findings

In this systematic review of 57 studies, for the association between vulvovaginal yeast infection and preterm birth the summary OR was 1.01 (95% CI 0.84–1.21,* I*^2^ 60%, prediction interval 0.45–2.23, 32 studies). For studies including ≥ 50% participants with symptoms the summary OR was 1.44 (95% CI 0.92–2.26, *I*^*2*^ 0%, two studies), studies with < 50% participants with symptoms 0.84 (95% CI 0.45–1.58, *I*^*2*^ 88%, seven studies) and studies including women without symptoms 1.12 (95% CI 0.94–1.35, *I*^*2*^ 0%, four studies). Most studies had a high risk of bias in at least one domain.

### Strengths and limitations

This review has several strengths. We had a broad search strategy with no language exclusion. By contacting authors, we obtained data that were not reported in the original publication and which added to this review (2 studies) [45, 79]. We pre-specified the analysis according to symptom status and described reporting and its association with preterm birth in detail. The main limitations of the review result from the high risk of bias and incomplete reporting of symptoms, which affect the certainty with which the findings can be interpreted. A methodological limitation is that only about 10% of the screened title and abstracts were verified by a second reviewer. Since this resulted in less than 3% additional studies for full-text screening, we decided that it is unlikely that the missed articles would have changed the results. Another methodological limitation is that, instead of two people extracting data independently, a second person verified the extracted data, which sped up the process but makes errors in the extracted data more likely.

### Comparison with other studies and interpretation

We found two relevant systematic reviews. Our findings are consistent with those of Schuster et al., who combined 15 studies and found no association between pregnant women with asymptomatic *Candida* colonisation and preterm birth (OR 1.10, 95% CI 0.99–1.22) [13]. In our review, we found that 11 studies in the meta-analysis by Schuster et al. included some women with symptoms of yeast infection, or in which the presence or absence of symptoms was not reported [45, 46, 49, 50, 60, 61, 63, 67, 85–87]. The second systematic review examined the effect of screening and treating women for asymptomatic vulvovaginal candidiasis during pregnancy [88]. In their meta-analysis of two randomised control trials [82, 83], women who received clotrimazole for diagnosed vaginal *Candida* were less likely to have a spontaneous preterm birth than women who received usual care (risk ratio 0.36, 95% CI 0.17–0.75). It is not clear why treatment of asymptomatic *Candida* in pregnancy was associated with a reduction in preterm birth in these two randomised control trials when no association was found in observational studies in which potential confounders were controlled for. The authors of the intervention review called for caution in the interpretation of their findings because data from the largest trial, which dominated the meta-analysis, were from an unplanned subgroup analysis [82].

We expected to observe a stronger association between symptomatic vulvovaginal yeast infections and preterm birth than for asymptomatic colonisation because we assumed increased inflammation in women with symptomatic infections [11]. The OR and the CI of the group with 50% or more symptomatic women are in the expected direction (OR 1.44, 95% CI 0.92–2.26) but the confidence intervals of the < 50% symptomatic and the group with no symptoms overlap and include one (OR 1.12, 95% CI 0.94–1.35). However, the quality of reporting in these studies varies and most studies were judged to have a high risk of bias in at least one domain. Despite this, the statistical heterogeneity between studies was mostly low. The three studies which provided an adjusted estimate also found ORs above one, in expected direction, but CIs were compatible with no association.

There are several possible reasons for the absence of an observed association in our meta-analyses of observational studies. First, the timepoint of testing during pregnancy varied in the assessed literature and most studies included only one timepoint; if women who had vulvovaginal yeast infections late in pregnancy, the onset of preterm labour would not be expected to be influenced [11]. Second, diagnostic factors might have played a role if testing was only done from the lower genital tract. If upper genital tract infection is a necessary precursor of preterm labour [4], test results from the upper genital tract would be needed. Third, different laboratory methods were used in the studies included in the review. Most included studies used culture which remains the gold standard, fewer used microscopy which is not as accurate as culture or polymerase chain reaction [89]. Even though we do not see a difference in the results when stratified by laboratory method used (Supplementary forest plots of stratified meta-analyses, Additional File 3), misclassification of the presence or absence of vulvovaginal yeast infection might have reduced the strength of association when studies were combined in meta-analysis. Fourth, treatment given differed between studies. Therefore, the effect of clearing of the infection versus persistent infection cannot be assessed.

## Conclusion

We systematically reviewed the literature about vulvovaginal yeast infections in pregnancy and adverse perinatal outcomes. We did not find strong statistical evidence of an increased risk for preterm birth or eight other adverse perinatal outcomes, in pregnant women with either symptomatic or asymptomatic vulvovaginal yeast infection. Further well-designed studies to collect detailed information about vulvovaginal yeast infections, symptom status and adverse pregnancy outcomes are warranted [89]. Use of molecular diagnostic methods would allow accurate detection and quantification of organism load to determine whether the presence of symptoms is associated with higher organism load, and whether higher organism loads are associated with a higher risk of preterm birth [9]. Finally, yeast infections cannot be seen in isolation and comprehensive evaluation of the role of concomitant vaginal or cervical infections, or certain microbiota should also be investigated in holistic studies [90–92]. Given the methodological limitations of observational studies and inconsistency with the findings of randomised controlled trials, there is insufficient evidence to make recommendations about testing and treatment of yeast infections in pregnancy to prevent preterm birth.

## Supplementary Information

**Additional file 1.** Search strategy. Search terms used for the literature search in eight databases.

**Additional file 2.** REDCap data extraction forms. Data extraction forms on REDCap which were designed and used to extract data from published articles for our systematic review.

**Additional file 3.** Forest plots of stratified meta-analyses. Forest plots of meta-analyses about vulvovaginal yeast infection and preterm birth stratified by study design, diagnostic method used, income setting, and time of testing.

**Additional file 4.** Forest plots of secondary outcomes. Forest plots of meta-analyses about vulvovaginal yeast infection and spontaneous abortion, stillbirth, preterm premature rupture of membranes, premature rupture of membranes, low birth weight, inflammation of the placenta or uterus.

**Additional file 5.** Summary of risk of bias assessment for cohort studies, cross-sectional studies, clinical trials, and case–control studies.

**Additional file 6.** Funnel plots of secondary outcomes preterm premature rupture of membranes and premature rupture of membranes.

## Data Availability

All data generated or analysed during this study are included in this published article, its supplementary information files or can be obtained via request to the corresponding author.

## Abbreviations

CI: Confidence interval
HIV: Human immunodeficiency virus
PROSPERO: International prospective register of systematic reviews
IQR: Interquartile range
OR: Odds ratio
PCR: Polymerase chain reaction
PPROM: Preterm premature rupture of membranes
PRISMA: Preferred reporting items for systematic reviews and meta-analyses
PROM: Premature rupture of membranes
REDCap: Research electronic data capture software
