# Supplementary material for: Vulvovaginal yeast infections during pregnancy and perinatal outcomes: systematic review and meta-analysis
Source: BMC Womens Health. 2023 Mar 21;23:116. doi: 10.1186/s12905-023-02258-7 (PMC10029297; doi:10.1186/s12905-023-02258-7)
Supplement: Supplementary file 1 — Additional file 1. Search strategy. Search terms used for the literature search in eight databases. [file 12905_2023_2258_MOESM1_ESM.docx]

**Additional file 1 - Search strategy**

**Medline Ovid (01.07.2022, 1922 records)**

| 1 exp pregnancy/ 972406  2 (pregnan* or gestation*).ti,ab. 669949  3 (labo?r or prelabo?r).ti,ab. 115368  4 obstetri*.ti,ab. 107118  5 parturition.ti,ab. 15331  6 (natal or prenatal or pre-natal or perinatal or peri-natal or postnatal or post-natal or postpartum or post-partum).ti,ab. 339780  7 or/1-6 1369811  8 exp Mycoses/ 135121  9 exp Candidiasis/ 33695  10 exp Yeasts/ 210686  11 exp Candida/ 49740  12 exp Saccharomyces/ 117073  13 exp Mycobiome/ 1012  14 exp RNA, Ribosomal, 18S/ 7336  15 mycos*.ti,ab. 18062  16 (candida or candidal or candidiasis or candidosis).ti,ab. 76647  17 monilia*.ti,ab. 1351  18 yeast*.ti,ab. 201609  19 saccharomyce*.ti,ab. 76918  20 (mycobiom* or mycobiot*).ti,ab. 1235  21 (18S adj3 (rRNA or RNA or sequenc*)).ti,ab. 9063  22 (ITS*1 adj3 sequenc*).ti,ab. 30835  23 internal* transcribed spacer*.ti,ab. 12796  24 or/8-23 484411  25 exp Pregnancy Outcome/ 82419  26 exp Pregnancy Complications/ 461281  27 exp Chorioamnionitis/ 3341  28 exp Endometritis/ 4305  29 exp Infant, Premature/ 62440  30 exp Infant, Low Birth Weight/ 37799  31 exp Fetal Growth Retardation/ 17928  32 exp Infant Mortality/ 31416  33 ((birth* or obstetric* or pregnan* or gestation*) adj3 (outcome* or complicat*)).ti,ab. 85236  34 ((natal or neonat* or newborn* or f?etal or fetal or f?etus or fetus or intrauterin* or intra-uterin* or "in utero" or infant* or antenatal or ante-natal or prenatal or pre-natal or perinatal or peri-natal or postnatal or post-natal or postpartum or post-partum) and (morbidit* or mortalit* or outcome* or death* or surviv*)).ti,ab. 301744  35 (premature* or pre-mature* or preterm* or pre-term*).ti,ab. 217638  36 (miscarriage* or abort*).ti,ab. 95836  37 (stillbirth* or still birth* or stillborn* or still born* or born-still*).ti,ab. 17823  38 (chorioamnionitis or funisitis or villitis or endometritis).ti,ab. 8927  39 (birth weight* or birthweight* or underweight*).ti,ab. 96258  40 ((f?etal or f?etus or intrauterin* or intra-uterin* or "in utero" or prenatal or pre-natal) adj3 growth*).ti,ab. 35634  41 small for gestation* age.ti,ab. 12049  42 or/25-41 962731  43 7 and 24 and 42 2725  44 exp animals/ not humans/ 5023312  45 43 not 44 2406  46 (congress or abstracts or case reports).pt. 2344143  47 45 not 46 1922 |
| --- |

**PubMed (01.07.2022, 2018 records)**

| 1 pregnancy[mh]  2 pregnan*[tiab] or gestation*[tiab]  3 labor[tiab] OR prelabor[tiab] OR labour[tiab] OR prelabour[tiab]  4 obstetri*[tiab]  5 parturition[tiab]  6 natal[tiab] OR prenatal[tiab] OR pre-natal[tiab] OR perinatal[tiab] OR peri-natal[tiab] OR postnatal[tiab] OR post-natal[tiab] OR postpartum[tiab] OR post-partum[tiab]  7 #1 OR #2 OR #3 OR #4 OR #5 OR #6  8 Mycoses[mh]  9 Candidiasis[mh]  10 Yeasts[mh]  11 Candida[mh]  12 Saccharomyces[mh]  13 Mycobiome[mh]  14 RNA, Ribosomal, 18S[mh]  15 mycos*[tiab]  16 candida[tiab] or candidal[tiab] or candidiasis[tiab] or candidosis[tiab]  17 monilia*[tiab]  18 yeast*[tiab]  19 saccharomyce*[tiab]  20 mycobiom*[tiab] or mycobiot*[tiab]  21 (18S[tiab] AND (rRNA[tiab] or RNA[tiab] or sequenc*[tiab]))  22 ((ITS[tiab] OR ITS1[tiab] OR ITS2[tiab] OR ITS4[tiab] OR ITS5[tiab]) AND sequenc*[tiab])  23 internal* transcribed spacer*[tiab]  24 #8 OR #9 OR #10 OR #11 OR #12 OR #13 OR #14 OR #15 OR #16 OR #17 OR #18 OR #19 OR #20 OR #21 OR #22 OR #23  25 Pregnancy Outcome[mh]  26 Pregnancy Complications[mh]  27 Chorioamnionitis[mh]  28 Endometritis[mh]  29 Infant, Premature[mh]  30 Infant, Low Birth Weight[mh]  31 Fetal Growth Retardation[mh]  32 Infant Mortality[mh]  33 (birth*[tiab] or obstetric*[tiab] or pregnan*[tiab] or gestation*[tiab]) AND (outcome*[tiab] or complicat*[tiab])  34 ((natal[tiab] or neonat*[tiab] or newborn*[tiab] or foetal[tiab] or fetal[tiab] or foetus[tiab] or fetus[tiab] or intrauterin*[tiab] or intra-uterin*[tiab] or "in utero"[tiab] or infant*[tiab] or antenatal[tiab] or ante-natal[tiab] or prenatal[tiab] or pre-natal[tiab] or perinatal[tiab] or peri-natal[tiab] or postnatal[tiab] or post-natal[tiab] or postpartum[tiab] or post-partum[tiab]) AND (morbidit*[tiab] or mortalit*[tiab] or outcome*[tiab] or death*[tiab] or surviv*[tiab]))  35 premature*[tiab] or pre-mature*[tiab] or preterm*[tiab] or pre-term*[tiab]  36 miscarriage*[tiab] or abort*[tiab]  37 stillbirth*[tiab] or still birth*[tiab] or stillborn*[tiab] or still born*[tiab] or born-still*[tiab]  38 chorioamnionitis[tiab] or funisitis[tiab] or villitis[tiab] or endometritis[tiab]  39 birth weight*[tiab] or birthweight*[tiab] or underweight*[tiab]  40 ((foetal[tiab] or fetal[tiab] or foetus[tiab] or fetus[tiab] or intrauterin*[tiab] or intra-uterin*[tiab] or "in utero"[tiab] or prenatal[tiab] or pre-natal[tiab]) AND (growth*[tiab]))  41 small for gestation*[tiab]  42 #25 OR #26 OR #27 OR #28 OR #29 OR #30 OR #31 OR #32 OR #33 OR #34 OR #35 OR #36 OR #37 OR #38 OR #39 OR #40 OR #41  43 #7 AND #24 AND #42  44 animals[mh] NOT humans[mh]  45 #43 NOT #44  46 congress[pt] or abstracts[pt] or case reports[pt]  47 #45 NOT #46 (2018) |
| --- |

**Embase Ovid (01.07.2022, 2067 records)**

| 1 exp pregnancy/ or pregnant woman/ or exp childbirth/ 806874  2 (pregnan* or gestation*).ti,ab. 858464  3 (labo?r or prelabo?r).ti,ab. 137723  4 obstetri*.ti,ab. 152196  5 parturition.ti,ab. 16880  6 (natal or prenatal or pre-natal or perinatal or peri-natal or postnatal or post-natal or postpartum or post-partum).ti,ab. 434439  7 or/1-6 1450878  8 exp mycosis/ 205934  9 exp candidiasis/ 53153  10 exp yeast/ 76904  11 exp Candida/ 92999  12 exp Saccharomyces/ 106976  13 mycobiome/ 1266  14 RNA 18S/ 10596  15 mycos*.ti,ab. 21781  16 (candida or candidal or candidiasis or candidosis).ti,ab. 97396  17 monilia*.ti,ab. 590  18 yeast*.ti,ab. 216407  19 saccharomyce*.ti,ab. 80609  20 (mycobiom* or mycobiot*).ti,ab. 1383  21 (18S adj3 (rRNA or RNA or sequenc*)).ti,ab. 9677  22 (ITS*1 adj3 sequenc*).ti,ab. 31524  23 internal* transcribed spacer*.ti,ab. 11273  24 or/8-23 583965  25 pregnancy outcome/ 72445  26 exp pregnancy complication/ or exp pregnancy disorder/ 595473  27 exp chorioamnionitis/ 10257  28 endometritis/ 7182  29 prematurity/ 116934  30 exp low birth weight/ 69952  31 exp intrauterine growth retardation/ 50827  32 infant mortality/ 21149  33 ((birth* or obstetric* or pregnan* or gestation*) adj3 (outcome* or complicat*)).ti,ab. 125134  34 ((natal or neonat* or newborn* or f?etal or fetal or f?etus or fetus or intrauterin* or intra-uterin* or "in utero" or infant* or antenatal or ante-natal or prenatal or pre-natal or perinatal or peri-natal or postnatal or post-natal or postpartum or post-partum) and (morbidit* or mortalit* or outcome* or death* or surviv*)).ti,ab. 412244  35 (premature* or pre-mature* or preterm* or pre-term*).ti,ab. 292610  36 (miscarriage* or abort*).ti,ab. 115954  37 (stillbirth* or still birth* or stillborn* or still born* or born-still*).ti,ab. 24203  38 (chorioamnionitis or funisitis or villitis or endometritis).ti,ab. 12704  39 (birth weight* or birthweight* or underweight*).ti,ab. 131886  40 ((f?etal or f?etus or intrauterin* or intra-uterin* or "in utero" or prenatal or pre-natal) adj3 growth*).ti,ab. 49526  41 small for gestation* age.ti,ab. 16426  42 or/25-41 1144273  43 7 and 24 and 42 3274  44 (exp animal/ or nonhuman/) not exp human/ 6847286  45 43 not 44 2977  46 conference abstract.pt. or case report/ 6919974  47 45 not 46 2067 |
| --- |

**Cochrane Library (01.07.2022, 172 results)**

[*Cochrane* Database of Systematic **Reviews**](https://www.cochranelibrary.com/) Issue 7 of 12, July 2022 (01.07.2022, **8 records)**

*Cochrane* Central Register of Controlled **Trials** Issue 7 of 12, July 2022 (01.07.2022, **164 records)**

ID Search Hits

1 (pregnan* or gestation*):ti,ab 72915

2 (labo*r or prelabo*r):ti,ab 15151

3 obstetri*:ti,ab 9819

4 parturition:ti,ab 230

5 (natal or prenatal or pre-natal or perinatal or peri-natal or postnatal or post-natal or postpartum or post-partum):ti,ab 22443

6 {OR 1-5} 92223

7 mycos*:ti,ab 1075

8 (candida or candidal or candidiasis or candidosis):ti,ab 3009

9 monilia*:ti,ab 33

10 yeast*:ti,ab 1695

11 saccharomyce*:ti,ab 471

12 (mycobiom* or mycobiot*):ti,ab 19

13 (18S NEAR/3 (rRNA or RNA or sequenc*)):ti,ab 24

14 ((ITS or ITS1 or ITS2 or ITS4 or ITS5) NEAR/3 sequenc*):ti,ab 83

15 (internal* transcribed spacer*):ti,ab 30

16 {OR 7-5} 5929

17 ((birth* or obstetric* or pregnan* or gestation*) NEAR/3 (outcome* or complicat*)):ti,ab 11969

18 ((natal or neonat* or newborn* or f*etal or fetal or f*etus or fetus or intrauterin* or intra-uterin* or in NEXT utero or infant* or antenatal or ante-natal or prenatal or pre-natal or perinatal or peri-natal or postnatal or post-natal or postpartum or post-partum) AND (morbidit* or mortalit* or outcome* or death* or surviv*)):ti,ab 41657

19 (premature* or pre-mature* or preterm* or pre-term*):ti,ab 28385

20 (miscarriage* or abort*):ti,ab 7157

21 (stillbirth* or still birth* or stillborn* or still born* or born-still*):ti,ab 2555

22 (chorioamnionitis or funisitis or villitis or endometritis):ti,ab 1315

23 (birth weight* or birthweight* or underweight*):ti,ab 14346

24 ((f*etal or f*etus or intrauterin* or intra-uterin* or in NEXT utero or prenatal or pre-natal) NEAR/3 growth*):ti,ab 1883

25 (small for gestation* age):ti,ab 1722

26 {OR 17-25} 74515

27 6 AND 16 AND 26 172

**CINAHL EBSCOhost (01.07.2022, 437 records)**

Search Screen - Advanced Search

Database - CINAHL with Full Text

Limiters/Expanders - Apply equivalent subjects; Search modes - Find all my search terms

S1 (MH "Pregnancy+") 236,838

S2 (MH "Expectant Mothers") 11,440

S3 (MH "Labor+") 15,203

S4 (MH "Gestational Age") 22,609

S5 (MH "Pregnancy Trimesters+") 13,317

S6 TI ( pregnan* or gestation* ) OR AB ( pregnan* or gestation* ) 187,392

S7 TI ( labour or labor or prelabour or prelabor ) OR AB ( labour or labor or prelabour or prelabor ) 41,227

S8 TI obstetri* OR AB obstetri* 37,112

S9 TI parturition OR AB parturition 845

S10 TI ( natal or prenatal or pre-natal or perinatal or peri-natal or postnatal or post-natal or postpartum or post-partum ) OR AB ( natal or prenatal or pre-natal or perinatal or peri-natal or postnatal or post-natal or postpartum or post-partum ) 92,556

S11 S1 OR S2 OR S3 OR S4 OR S5 OR S6 OR S7 OR S8 OR S9 OR S10 363,852

S12 (MH "Mycoses+") 18,811

S13 (MH "Candidiasis+") 5,086

S14 (MH "Yeasts+") 5,471

S15 (MH "Candida") 2,029

S16 (MH "Saccharomyces") 85

S17 TI mycos* OR AB mycos* 1,438

S18 TI ( candida or candidal or candidiasis or candidosis ) OR AB ( candida or candidal or candidiasis or candidosis ) 7,673

S19 TI monilia* OR AB monilia* 37

S20 TI yeast* OR AB yeast* 3,709

S21 TI saccharomyce* OR AB saccharomyce* 659

S22 TI ( mycobiom* or mycobiot* ) OR AB ( mycobiom* or mycobiot* ) 73

S23 TI ( (18S N3 (rRNA or RNA or sequenc*)) ) OR AB ( (18S N3 (rRNA or RNA or sequenc*)) ) 114

S24 TI ITS# N3 sequenc* OR AB ITS# N3 sequenc* 542

S25 TI internal* transcribed spacer* OR AB internal* transcribed spacer* 250

S26 S12 OR S13 OR S14 OR S15 OR S16 OR S17 OR S18 OR S19 OR S20 OR S21 OR S22 OR S23 OR S24 OR S25 28,124

S27 (MH "Pregnancy Outcomes") 27,192

S28 (MH "Pregnancy Complications+") 105,039

S29 (MH "Chorioamnionitis") 1,360

S30 (MH "Infant, Premature") 25,716

S31 (MH "Infant, Low Birth Weight+") 15,814

S32 (MH "Fetal Growth Retardation") 5,073

S33 (MH "Infant Mortality") 9,965

S34 TI ( ((birth* or obstetric* or pregnan* or gestation*) N3 (outcome* or complicat*)) ) OR AB ( ((birth* or obstetric* or pregnan* or gestation*) N3 (outcome* or complicat*)) ) 35,498

S35 TI ( ((natal or neonat* or newborn* or foetal or fetal or foetus or fetus or intrauterin* or intra-uterin* or "in utero" or infant* or antenatal or ante-natal or prenatal or pre-natal or perinatal or peri-natal or postnatal or post-natal or postpartum or post-partum) AND (morbidit* or mortalit* or outcome* or death* or surviv*)) ) OR AB ( ((natal or neonat* or newborn* or foetal or fetal or foetus or fetus or intrauterin* or intra-uterin* or "in utero" or infant* or antenatal or ante-natal or prenatal or pre-natal or perinatal or peri-natal or postnatal or post-natal or postpartum or post-partum) AND (morbidit* or mortalit* or outcome* or death* or surviv*)) ) 92,182

S36 TI ( premature* or pre-mature* or preterm* or pre-term* ) OR AB ( premature* or pre-mature* or preterm* or pre-term* ) 63,797

S37 TI ( miscarriage* or abort* ) OR AB ( miscarriage* or abort* ) 18,922

S38 TI ( stillbirth* or still-birth* or stillborn* or still-born* or born-still* ) OR AB ( stillbirth* or still-birth* or stillborn* or still-born* or born-still* ) 5,833

S39 TI ( chorioamnionitis or funisitis or villitis or endometritis ) OR AB ( chorioamnionitis or funisitis or villitis or endometritis ) 2,308

S40 TI ( birth N1 weight* or birthweight* or underweight* ) OR AB ( birth N1 weight* or birthweight* or underweight* ) 33,053

S41 TI ( ((fetal or foetal or fetus or foetus or intrauterin* or intra-uterin* or "in utero" or prenatal or pre-natal) N3 growth*) ) OR AB ( ((fetal or foetal or fetus or foetus or intrauterin* or intra-uterin* or "in utero" or prenatal or pre-natal) N3 growth*) ) 9,563

S42 TI small for gestation* age OR AB small for gestation* age 6,331

S43 S27 OR S28 OR S29 OR S30 OR S31 OR S32 OR S33 OR S34 OR S35 OR S36 OR S37 OR S38 OR S39 OR S40 OR S41 OR S42 256,980

S44 S11 AND S26 AND S43 437

**African Index Medicus** (via Global Index Medicus) **(01.07.2022, 15 records)**

tw:(((candid* OR yeast* OR monilia* OR mycobiom* OR mycos*) AND (pregnan* OR preterm OR premature OR miscarriage* OR abort* OR stillbirth* OR still-birth* OR stillborn*)) ) AND ( collection_gim:("AIM"))

**LILACS** (via Global Index Medicus) **(01.07.2022, 271 records)
Latin American and Caribbean Center on Health Sciences Information**

tw:((("candida" OR candidias* OR candidos* OR yeast* OR monilia* OR mycobiom* OR mycos*) AND (pregnan* OR preterm OR premature OR miscarriage* OR abort* OR stillbirth* OR still-birth* OR stillborn*)) ) AND ( collection_gim:("LILACS"))

**ClinicalTrials.gov (01.07.2022, 55 studies)**

**Expert Search:**

(( candida OR candidias* OR candidos* OR yeast* OR monilia* OR mycobiom* OR mycos* OR fungal ) AND ( pregnan* OR preterm OR premature OR miscarriage* OR abort* OR stillbirth* OR still-birth* OR stillborn* ))
