# Supplementary material for: Vulvovaginal yeast infections during pregnancy and perinatal outcomes: systematic review and meta-analysis
Source: BMC Womens Health. 2023 Mar 21;23:116. doi: 10.1186/s12905-023-02258-7 (PMC10029297; doi:10.1186/s12905-023-02258-7)
Supplement: Supplementary file 2 — Additional file 2. REDCap data extraction forms. Data extraction forms on REDCap which were designed and used to extract data from published articles for our systematic review. [file 12905_2023_2258_MOESM2_ESM.pdf]

Screening Form

Record ID	________________________________
Article upload	
Last name, initial of first author	________________________________
Year of publication	________________________________
Include after review of full text?	◯ Yes ◯ No
What was the primary reason for excluding the text?	◯ Article is a review<br>◯ Article is a case report/series<br>◯ Article is an opinion piece/commentary/editorial<br>◯ Infection not microbiologically confirmed<br>◯ No vaginal specimen taken for the detection of yeast<br>◯ Control group missing<br>◯ None of the outcomes of interest are included<br>◯ Does not report relevant numerical data<br>◯ Full text not found<br>◯ Other (please specify below)
Other reason for exclusion	____
Please enter any additional comments:	____
Screened by?	________________________________<br>(Enter initials only.)
Verified by?	________________________________<br>(Enter initials only.)

Data Extraction Form (Study and population characteristics)

Study design	◯ Case-control<br>◯ Cross-sectional<br>◯ Cohort<br>◯ Clinical trial<br>◯ Other (please specify below)
Other study design	__________________________________

Study Country

- ◯ Afghanistan
- ◯ Albania
- ◯ Algeria
- ◯ Andorra
- ◯ Angola
- ◯ Antigua and Barbuda
- ◯ Argentina
- ◯ Armenia
- ◯ Australia
- ◯ Austria
- ◯ Azerbaijan
- ◯ Bahamas
- ◯ Bahrain
- ◯ Bangladesh
- ◯ Barbados
- ◯ Belarus
- ◯ Belgium
- ◯ Belize
- ◯ Benin
- ◯ Bhutan
- ◯ Bolivia
- ◯ Bosnia and Herzegovina
- ◯ Botswana
- ◯ Brazil
- ◯ Brunei
- ◯ Bulgaria
- ◯ Burkina Faso
- ◯ Burundi
- ◯ Cabo Verde
- ◯ Cambodia
- ◯ Cameroon
- ◯ Canada
- ◯ Central African Republic (CAR)
- ◯ Chad
- ◯ Chile
- ◯ China
- ◯ Colombia
- ◯ Comoros
- ◯ Congo (Democratic Republic of the Congo)
- ◯ Congo (Republic of the Congo)
- ◯ Costa Rica
- ◯ Cote d'Ivoire
- ◯ Croatia
- ◯ Cuba
- ◯ Cyprus
- ◯ Czechia
- ◯ Denmark
- ◯ Djibouti
- ◯ Dominica
- ◯ Dominican Republic
- ◯ Ecuador
- ◯ Egypt
- ◯ El Salvador
- ◯ Equatorial Guinea
- ◯ Eritrea
- ◯ Estonia
- ◯ Eswatini (formerly Swaziland)
- ◯ Ethiopia
- ◯ Fiji
- ◯ Finland
- ◯ France
- ◯ Gabon
- ◯ Gambia
- ◯ Georgia
- ◯ Germany
- ◯ Ghana
- ◯ Greece
- ◯ Grenada
- ◯ Guatemala

◯ Guinea
◯ Guinea-Bissau
◯ Guyana
◯ Haiti
◯ Honduras
◯ Hungary
◯ Iceland
◯ India
◯ Indonesia
◯ Iran
◯ Iraq
◯ Ireland
◯ Israel
◯ Italy
◯ Jamaica
◯ Japan
◯ Jordan
◯ Kazakhstan
◯ Kenya
◯ Kiribati
◯ Kosovo
◯ Kuwait
◯ Kyrgyzstan
◯ Laos
◯ Latvia
◯ Lebanon
◯ Lesotho
◯ Liberia
◯ Libya
◯ Liechtenstein
◯ Lithuania
◯ Luxembourg
◯ Madagascar
◯ Malawi
◯ Malaysia
◯ Maldives
◯ Mali
◯ Malta
◯ Marshall Islands
◯ Mauritania
◯ Mauritius
◯ Mexico
◯ Micronesia
◯ Moldova
◯ Monaco
◯ Mongolia
◯ Montenegro
◯ Morocco
◯ Mozambique
◯ Myanmar (formerly Burma)
◯ Namibia
◯ Nauru
◯ Nepal
◯ Netherlands
◯ New Zealand
◯ Nicaragua
◯ Niger
◯ Nigeria
◯ North Korea
◯ North Macedonia (formerly Macedonia)
◯ Norway
◯ Oman
◯ Pakistan
◯ Palau
◯ Palestine
◯ Panama
◯ Papua New Guinea
◯ Paraguay
◯ Peru
◯ Philippines
◯ Poland

◯ Portugal
◯ Qatar
◯ Romania
◯ Russia
◯ Rwanda
◯ Saint Kitts and Nevis
◯ Saint Lucia
◯ Saint Vincent and the Grenadines
◯ Samoa
◯ San Marino
◯ Sao Tome and Principe
◯ Saudi Arabia
◯ Senegal
◯ Serbia
◯ Seychelles
◯ Sierra Leone
◯ Singapore
◯ Slovakia
◯ Slovenia
◯ Solomon Islands
◯ Somalia
◯ South Africa
◯ South Korea
◯ South Sudan
◯ Spain
◯ Sri Lanka
◯ Sudan
◯ Suriname
◯ Sweden
◯ Switzerland
◯ Syria
◯ Taiwan
◯ Tajikistan
◯ Tanzania
◯ Thailand
◯ Timor-Leste
◯ Togo
◯ Tonga
◯ Trinidad and Tobago
◯ Tunisia
◯ Turkey
◯ Turkmenistan
◯ Tuvalu
◯ Uganda
◯ Ukraine
◯ United Arab Emirates (UAE)
◯ United Kingdom (UK)
◯ United States of America (USA)
◯ Uruguay
◯ Uzbekistan
◯ Vanuatu
◯ Vatican City (Holy See)
◯ Venezuela
◯ Vietnam
◯ Yemen
◯ Zambia
◯ Zimbabwe
◯ Other
◯ Multiple
◯ not reported
(if more than one, choose multiple)

Other or multiple study country

____
(Separate variables with a comma.)

Study Aim	______________________ (If missing, enter "99999".)
In which year did the study begin?	______________________ (Year given by the author. Can be study start/enrolment start/data collection start or the like. If missing, enter "99999".)
In which year did the study finish?	______________________ (Year given by the author. Can be study end/enrolment completion/data collection completion/end of follow-up or the like. If missing, enter "99999".)
Number of women invited	______________________ (If missing, enter "99999".)
Number of women eligible	______________________ (If missing, enter "99999".)
Number of women enrolled	______________________ (If missing, enter "99999".)
How was gestational age measured for all women?	☐ Ultrasound<br>☐ Fundal height<br>☐ Last menstrual period<br>☐ Newborn assessment<br>☐ Mixed methods<br>☐ Not reported/unclear
Main inclusion criteria	______________________ (If missing, enter "99999".)
How is age reported?	☐ Mean<br>☐ Mean with STD<br>☐ Median<br>☐ Median with IQR<br>☐ Median with STD<br>☐ Age range<br>☐ Other (please specify below)<br>☐ Not reported/unclear
Mean age: Is age reported separately for each study group?	◯ Yes<br>◯ No
Mean age: For which group is age reported separately?	◯ Vaginal-yeast infection<br>◯ Outcome<br>◯ Other (please specify below)

Mean age: Other group for which age is reported separately	__________________________________
Mean age	__________________________________
Mean age STD	__________________________________
Mean age for positive group (for example with vaginal-yeast infection/with outcome)	__________________________________
Mean age STD for positive group (for example with vaginal-yeast infection/with outcome)	__________________________________
Mean age for negative group (for example without vaginal-yeast infection/without outcome)	__________________________________
Mean age STD for negative group (for example without vaginal-yeast infection/without outcome)	__________________________________
Median age: Is age reported separately for each study group?	○ Yes<br>○ No
Median age: For which group is age reported separately?	○ Vaginal-yeast infection<br>○ Outcome<br>○ Other (please specify below)
Median age: Other group for which age is reported separately	__________________________________
Median age	__________________________________
Median age IQR: What is the value for the lower IQR?	__________________________________
Median age IQR: What is the value for the higher IQR?	__________________________________
Median age STD	__________________________________
Median age for positive group (for example with vaginal-yeast infection/with outcome)	__________________________________
Median age IQR for positive group (for example with vaginal-yeast infection/with outcome): What is the value for the lower limit for IQR?	__________________________________
Median age IQR for positive group (for example with vaginal-yeast infection/with outcome): What is the value for the higher limit for IQR?	__________________________________
Median age STD for positive group (for example with vaginal-yeast infection/with outcome)	__________________________________

Median age for negative group (for example without vaginal-yeast infection/without outcome)	__________________________________
Median age IQR for negative group (for example without vaginal-yeast infection/without outcome): What is the value for the lower limit for IQR?	__________________________________
Median age IQR for negative group (for example without vaginal-yeast infection/without outcome): What is the value for the higher limit for IQR?	__________________________________
Median age STD for negative group (for example without vaginal-yeast infection/without outcome)	__________________________________
Age range: Is age reported separately for each study group?	◯ Yes<br>◯ No
Age range: For which group is age reported separately?	◯ Vaginal-yeast infection<br>◯ Outcome<br>◯ Other (please specify below)
Age range: Other group for which age is reported separately	__________________________________
Minimum age	__________________________________
Maximum age	__________________________________
Minimum age for positive group (for example with vaginal-yeast infection/with outcome)	__________________________________
Maximum age for positive group (for example with vaginal-yeast infection/with outcome)	__________________________________
Minimum age for negative group (for example without vaginal-yeast infection/without outcome)	__________________________________
Maximum age for negative group (for example without vaginal-yeast infection/without outcome)	__________________________________
Other age report	____
Does the study include women with signs and symptoms of vaginal candida?	◯ Yes<br>◯ No<br>◯ Not reported/unclear<br>(Signs: vulvo-vaginitis, curdy white discharge / Symptoms: vulval itchiness, discharge)
Did the majority of women included in the study have signs and/or symptoms?	◯ Yes<br>◯ No<br>◯ Not reported/unclear

What is the proportion (%) of women with signs and symptoms of vaginal candida included in the study?	______________________<br>(If missing, enter "99999" / Signs: vulvo-vaginitis, curdy white discharge / Symptoms: vulval itchiness, discharge)
Is the proportion (%) of women with signs and symptoms of vaginal candida reported separately for infection status and/or outcome?	◯ Yes<br>◯ No
Does the study include women with diabetes?	◯ Yes<br>◯ No<br>◯ Not reported/unclear
What is the proportion (%) of women with diabetes included in the study?	______________________<br>(If missing, enter "99999")
Does the study include smokers?	◯ Yes<br>◯ No<br>◯ Not reported/unclear
What is the proportion (%) of smokers included in the study?	______________________<br>(If missing, enter "99999")
Does the study include women with multiple pregnancies (e.g.<br>twins, triplets)?	◯ Yes<br>◯ No<br>◯ Not reported/unclear
What is the proportion (%) of women with multiple pregnancies included in the study?	______________________<br>(If missing, enter "99999")
Does the study include patients with HIV/AIDS?	◯ Yes<br>◯ No<br>◯ Not reported/unclear
What is the proportion (%) of patients with HIV/AIDS included in the study?	______________________<br>(If missing, enter "99999")
Are other genital infections reported?	◯ Yes<br>◯ No<br>◯ Not reported/unclear

Other reported genital infections	☐ Bacterial vaginosis<br>☐ Chlamydia trachomatis<br>☐ Trichomonas vaginalis<br>☐ Microbiome/Mycobiome<br>☐ Neisseria gonorrhoeae<br>☐ Mycoplasma genitalium<br>☐ Mycoplasma hominis<br>☐ Gardnerella vaginalis<br>☐ Ureaplasma urealyticum<br>☐ Ureaplasma parvum<br>☐ GBS<br>☐ Bacteroides<br>☐ Lactobacillus<br>☐ Other (please specify below)<br>☐ Not reported/unclear
Other reported infections (please specify)	__________________________________<br>(Separate variables with a comma.)
Please enter any additional comments	__________________________________
Extracted by?	____________________________<br>(Enter initials only.)
Verified by?	____________________________<br>(Enter initials only.)

Data Extraction Form (Lab characteristics)

Specimen type	☐ Vaginal swab<br>☐ Endocervical swab<br>☐ Other (please specify below)<br>☐ Not reported/unclear
Specify other specimen type	__________________________________
Specimen collection method	○ Self collected<br>○ Clinican collected<br>○ Not reported/unclear
What was the gestational age at specimen collection?	☐ 1st trimester (0-13 weeks)<br>☐ 2nd trimester (14-26 weeks)<br>☐ 3rd trimester (27-40 weeks)<br>☐ Post-partum<br>☐ Not reported/unclear<br>(Tick all trimesters included)
Diagnostic tools used to detect yeast	☐ Wet preparation<br>☐ Gram stain<br>☐ Culture<br>☐ Microbiome/Mycobiome<br>☐ PCR<br>☐ Other (please specify below)<br>☐ Not reported/unclear
Specify other diagnostic tools used	__________________________________<br>(Separate variables with a comma.)
Yeast infection characteristics	☐ spores<br>☐ budding<br>☐ Not reported/unclear
Type of yeast infection	☐ C. albicans<br>☐ C. tropicalis<br>☐ C. glabrata<br>☐ C. parapsilosis<br>☐ C. krusei<br>☐ Other (please specify below)<br>☐ Not reported/unclear
Specify other yeast types	__________________________________<br>(Separate variables with a comma. If missing, enter "99999".)
Treatment history	☐ Antibiotic (please specify below)<br>☐ Antifungal (please specify below)<br>☐ Probiotics (please specify below)<br>☐ Other (please specify below)<br>☐ No treatment<br>☐ Not reported/unclear

Specify antibiotic treatment

____
(Separate variables with a comma. If missing, enter "99999".)

Specify antifungal treatment

____
(Separate variables with a comma. If missing, enter "99999".)

Specify probiotic treatment

____
(Separate variables with a comma. If missing, enter "99999".)

Specify other treatments

____
(Separate variables with a comma. If missing, enter "99999".)

What was the gestational age at treatment?

- ☐ 1st trimester (0-13 weeks)
- ☐ 2nd trimester (14-26 weeks)
- ☐ 3rd trimester (27-40 weeks)
- ☐ Post-partum
- ☐ Not reported/unclear

Please enter any additional comments

____

Extracted by?

____
(Enter initials only.)

Verified by?

____
(Enter initials only.)

Data Extraction Form (Outcome)

Outcomes of interest

- ☐ Preterm birth
- ☐ Miscarriage or spontaneous abortion
- ☐ Stillbirth including fetal death
- ☐ Preterm premature rupture of membranes
- ☐ Premature rupture of membranes
- ☐ Low birth weight
- ☐ Small for gestational age
- ☐ Inflammation of the placenta or uterus
- ☐ Neonatal death
- ☐ Neonatal fungal sepsis
- ☐ Other (please specify below)

Please specify other outcomes of interest

____
(Separate variables with a comma.)

Preterm birth

Is preterm birth defined as birth before 37 completed weeks of pregnancy?

- ◯ Yes
- ◯ No
- ◯ Not reported/unclear

Preterm birth is defined as less than how many weeks?

____
(If missing, enter "99999".)

Number of women included in preterm analysis

____
(If missing, enter "99999".)

Were outcomes reported separately for women with diabetes?

- ◯ Yes
- ◯ No

Were outcomes reported separately for co-infections?

- ◯ Yes
- ◯ No

Were outcomes reported separately for albicans and non-albicans species?

- ◯ Yes
- ◯ No

Preterm birth: Number with outcome and VY+

____
(If missing, enter "99999".)

Preterm birth: Number with outcome and VY-

____
(If missing, enter "99999".)

Preterm birth: Number without outcome and VY+

____
(If missing, enter "99999".)

Preterm birth: Number without outcome and VY-	______________ (If missing, enter "99999".)
Are the above numbers provided by the author or calculated from percentages?	◯ raw numbers provided<br>◯ calculated from percentage<br>◯ both
Preterm birth: Was a RR reported?	◯ Yes<br>◯ No
Preterm birth: The (RR) reported results were	◯ Adjusted<br>◯ Unadjusted<br>◯ Both<br>◯ Not reported/unclear
Preterm birth: Enter adjusted RR	______________
Preterm birth: Was a 95% CI reported for the adjusted RR?	◯ Yes<br>◯ No
Preterm birth: What is the value for the lower limit for the adjusted RR?	______________ (Report using two decimal places.)
Preterm birth: What is the value for the higher limit for the adjusted RR?	______________ (Report using two decimal places.)
Preterm birth: Enter variables adjusted for in analysis	______________ (Separate variables with a comma. If missing, enter "99999".)
Preterm birth: Enter unadjusted RR	______________
Preterm birth: Was a 95% CI reported for the unadjusted RR?	◯ Yes<br>◯ No
Preterm birth: What is the value for the lower limit for the unadjusted RR?	______________ (Report using two decimal places.)
Preterm Birth: What is the value for the higher limit for the unadjusted RR?	______________ (Report using two decimal places.)
Preterm birth: Was an OR reported?	◯ Yes<br>◯ No
Preterm birth: The (OR) reported results were	◯ Adjusted<br>◯ Unadjusted<br>◯ Both<br>◯ Not reported/unclear

Preterm birth: Enter adjusted OR

____

Preterm birth: Was a 95% CI reported for the adjusted OR?

◯ Yes
◯ No

Preterm birth: What is the value for the lower limit for the adjusted OR?

____
(Report using two decimal places.)

Preterm birth: What is the value for the higher limit for the adjusted OR?

____
(Report using two decimal places.)

Preterm birth: Enter variables adjusted for in analysis

____
(Separate variables with a comma. If missing, enter "99999".)

Preterm birth: Enter unadjusted OR

____

Preterm birth: Was a 95% CI reported for the unadjusted OR?

◯ Yes
◯ No

Preterm birth: What is the value for the lower limit for the unadjusted OR?

____
(Report using two decimal places.)

Preterm birth: What is the value for the higher limit for the unadjusted OR?

____
(Report using two decimal places.)

Preterm birth: Was an HR reported?

◯ Yes
◯ No

Preterm birth: The (HR) reported results were

◯ Adjusted
◯ Unadjusted
◯ Both
◯ Not reported/unclear

Preterm birth: Enter adjusted HR

____

Preterm birth: Was a 95% CI reported for the adjusted HR?

◯ Yes
◯ No

Preterm birth: What is the value for the lower limit for the adjusted HR?

____
(Report using two decimal places.)

Preterm birth: What is the value for the higher limit for the adjusted HR?

____
(Report using two decimal places.)

Preterm birth: Enter variables adjusted for in analysis

____
(Separate variables with a comma. If missing, enter "99999".)

Preterm birth: Enter unadjusted HR

____

Preterm birth: Was a 95% CI reported for the unadjusted HR?

- ◯ Yes
- ◯ No

Preterm birth: What is the value for the lower limit for the unadjusted HR?

____
(Report using two decimal places.)

Preterm birth: What is the value for the higher limit for the unadjusted HR?

____
(Report using two decimal places.)

Misscarriage or spontaneous abortion

Miscarriage or spontaneous abortion defined as delivery of dead fetus before 22 completed weeks of pregnancy?

- ◯ Yes
- ◯ No
- ◯ Not reported/unclear

Please specify definition of miscarriage or spontaneous abortion

____
(If missing, enter "99999".)

Number of women included in spontaneous abortion analysis

____
(If missing, enter "99999".)

Were outcomes reported separately for women with diabetes?

- ◯ Yes
- ◯ No

Were outcomes reported separately for co-infections?

- ◯ Yes
- ◯ No

Were outcomes reported separately for albicans and non-albicans species?

- ◯ Yes
- ◯ No

Spontaneous abortion: Number with outcome and VY+

____
(If missing, enter "99999".)

Spontaneous abortion: Number with outcome and VY-

____
(If missing, enter "99999".)

Spontaneous abortion: Number without outcome and VY+

____
(If missing, enter "99999".)

Spontaneous abortion: Number without outcome and VY-	______________________ (If missing, enter "99999".)
Are the above numbers provided by the author or calculated from percentages?	◯ raw numbers provided<br>◯ calculated from percentage<br>◯ both
Spontaneous abortion: Was a RR reported?	◯ Yes<br>◯ No
Spontaneous abortion: The (RR) reported results were	◯ Adjusted<br>◯ Unadjusted<br>◯ Both<br>◯ Not reported/unclear
Spontaneous abortion: Enter adjusted RR	______________________
Spontaneous abortion: Was a 95% CI reported for the adjusted RR?	◯ Yes<br>◯ No
Spontaneous abortion: What is the value for the lower limit for the adjusted RR?	______________________ (Report using two decimal places.)
Spontaneous abortion: What is the value for the higher limit for the adjusted RR?	______________________ (Report using two decimal places.)
Spontaneous abortion: Enter variables adjusted for in analysis	______________________ (Separate variables with a comma. If missing, enter "99999".)
Spontaneous abortion: Enter unadjusted RR	______________________
Spontaneous abortion: Was a 95% CI reported for the unadjusted RR?	◯ Yes<br>◯ No
Spontaneous abortion: What is the value for the lower limit for the unadjusted RR?	______________________ (Report using two decimal places.)
Spontaneous abortion: What is the value for the higher limit for the unadjusted RR?	______________________ (Report using two decimal places.)
Spontaneous abortion: Was an OR reported?	◯ Yes<br>◯ No
Spontaneous abortion: The (OR) reported results were	◯ Adjusted<br>◯ Unadjusted<br>◯ Both<br>◯ Not reported/unclear

Spontaneous abortion: Enter adjusted OR	______________________
Spontaneous abortion: Was a 95% CI reported for the adjusted OR?	◯ Yes<br>◯ No
Spontaneous abortion: What is the value for the lower limit for the adjusted OR?	______________________<br>(Report using two decimal places.)
Spontaneous abortion: What is the value for the higher limit for the adjusted OR?	______________________<br>(Report using two decimal places.)
Spontaneous abortion: Enter variables adjusted for in analysis	______________________<br>(Separate variables with a comma. If missing, enter "99999".)
Spontaneous abortion: Enter unadjusted OR	______________________
Spontaneous abortion: Was a 95% CI reported for the unadjusted OR?	◯ Yes<br>◯ No
Spontaneous abortion: What is the value for the lower limit for the unadjusted OR?	______________________<br>(Report using two decimal places.)
Spontaneous abortion: What is the value for the higher limit for the unadjusted OR?	______________________<br>(Report using two decimal places.)
Spontaneous abortion: Was an HR reported?	◯ Yes<br>◯ No
Spontaneous abortion: The (HR) reported results were	◯ Adjusted<br>◯ Unadjusted<br>◯ Both<br>◯ Not reported/unclear
Spontaneous abortion: Enter adjusted HR	______________________
Spontaneous abortion: Was a 95% CI reported for the adjusted HR?	◯ Yes<br>◯ No
Spontaneous abortion: What is the value for the lower limit for the adjusted HR?	______________________<br>(Report using two decimal places.)
Spontaneous abortion: What is the value for the higher limit for the adjusted HR?	______________________<br>(Report using two decimal places.)

Spontaneous abortion: Enter variables adjusted for in analysis

____
(Separate variables with a comma. If missing, enter "99999".)

Spontaneous abortion: Enter unadjusted HR

____

Spontaneous abortion: Was a 95% CI reported for the unadjusted HR?
◯ Yes
◯ No

Spontaneous abortion: What is the value for the lower limit for the unadjusted HR?

____
(Report using two decimal places.)

Spontaneous abortion: What is the value for the higher limit for the unadjusted HR?

____
(Report using two decimal places.)

Stillbirth including fetal death

Stillbirth including fetal death defined as delivery of dead fetus after 22 completed weeks of pregnancy?
◯ Yes
◯ No
◯ Not reported/unclear

Please specify definition of stillbirth including fetal death

____

Number of women included in stillbirth analysis

____
(If missing, enter "99999".)

Were outcomes reported separately for women with diabetes?
◯ Yes
◯ No

Were outcomes reported separately for co-infections?
◯ Yes
◯ No

Were outcomes reported separately for albicans and non-albicans species?
◯ Yes
◯ No

Stillbirth: Number with outcome and VY+

____
(If missing, enter "99999".)

Stillbirth: Number with outcome and VY-

____
(If missing, enter "99999".)

Stillbirth: Number without outcome and VY+

____
(If missing, enter "99999".)

Stillbirth: Number without outcome and VY-	______________________ (If missing, enter "99999".)
Are the above numbers provided by the author or calculated from percentages?	◯ raw numbers provided<br>◯ calculated from percentage<br>◯ both
Stillbirth: Was a RR reported?	◯ Yes<br>◯ No
Stillbirth: The (RR) reported results were	◯ Adjusted<br>◯ Unadjusted<br>◯ Both<br>◯ Not reported/unclear
Stillbirth: Enter adjusted RR	______________________
Stillbirth: Was a 95% CI reported for the adjusted RR?	◯ Yes<br>◯ No
Stillbirth: What is the value for the lower limit for the adjusted RR?	______________________ (Report using two decimal places.)
Stillbirth: What is the value for the higher limit for the adjusted RR?	______________________ (Report using two decimal places.)
Stillbirth: Enter variables adjusted for in analysis	______________________ (Separate variables with a comma. If missing, enter "99999".)
Stillbirth: Enter unadjusted RR	______________________
Stillbirth: Was a 95% CI reported for the unadjusted RR?	◯ Yes<br>◯ No
Stillbirth: What is the value for the lower limit for the unadjusted RR?	______________________ (Report using two decimal places.)
Stillbirth: What is the value for the higher limit for the unadjusted RR?	______________________ (Report using two decimal places.)
Stillbirth: Was an OR reported?	◯ Yes<br>◯ No
Stillbirth: The (OR) reported results were	◯ Adjusted<br>◯ Unadjusted<br>◯ Both<br>◯ Not reported/unclear

Stillbirth: Enter adjusted OR	__________________________________
Stillbirth: Was a 95% CI reported for the adjusted OR?	◯ Yes<br>◯ No
Stillbirth: What is the value for the lower limit for the adjusted OR?	__________________________________<br>(Report using two decimal places.)
Stillbirth: What is the value for the higher limit for the adjusted OR?	__________________________________<br>(Report using two decimal places.)
Stillbirth: Enter variables adjusted for in analysis	____<br>(Separate variables with a comma. If missing, enter "99999".)
Stillbirth: Enter unadjusted OR	__________________________________
Stillbirth: Was a 95% CI reported for the unadjusted OR?	◯ Yes<br>◯ No
Stillbirth: What is the value for the lower limit for the unadjusted OR?	__________________________________<br>(Report using two decimal places.)
Stillbirth: What is the value for the higher limit for the unadjusted OR?	__________________________________<br>(Report using two decimal places.)
Stillbirth: Was an HR reported?	◯ Yes<br>◯ No
Stillbirth: The (HR) reported results were	◯ Adjusted<br>◯ Unadjusted<br>◯ Both<br>◯ Not reported/unclear
Stillbirth: Enter adjusted HR	__________________________________
Stillbirth: Was a 95% CI reported for the adjusted HR?	◯ Yes<br>◯ No
Stillbirth: What is the value for the lower limit for the adjusted HR?	__________________________________<br>(Report using two decimal places.)
Stillbirth: What is the value for the higher limit for the adjusted HR?	__________________________________<br>(Report using two decimal places.)

Stillbirth: Enter variables adjusted for in analysis

____
(Separate variables with a comma. If missing, enter "99999".)

Stillbirth: Enter unadjusted HR

____

Stillbirth: Was a 95% CI reported for the unadjusted HR?

- ◯ Yes
- ◯ No

Stillbirth: What is the value for the lower limit for the unadjusted HR?

____
(Report using two decimal places.)

Stillbirth: What is the value for the higher limit for the unadjusted HR?

____
(Report using two decimal places.)

Preterm premature rupture of membranes

Preterm premature rupture of membranes defined as spontaneous tearing of the membranes surrounding the fetus before 37 weeks of gestation?

- ◯ Yes
- ◯ No
- ◯ Not reported/unclear

Please specify definition of preterm premature rupture of membranes

____

Number of women included in PPROM analysis

____
(If missing, enter "99999".)

Were outcomes reported separately for women with diabetes?

- ◯ Yes
- ◯ No

Were outcomes reported separately for co-infections?

- ◯ Yes
- ◯ No

Were outcomes reported separately for albicans and non-albicans species?

- ◯ Yes
- ◯ No

PPROM: Number with outcome and VY+

____
(If missing, enter "99999".)

PPROM: Number with outcome and VY-

____
(If missing, enter "99999".)

PPROM: Number without outcome and VY+

____
(If missing, enter "99999".)

PPROM: Number without outcome and VY-	__________________________________ (If missing, enter "99999".)
Are the above numbers provided by the author or calculated from percentages?	◯ raw numbers provided<br>◯ calculated from percentage<br>◯ both
PPROM: Was a RR reported?	◯ Yes<br>◯ No
PPROM: The (RR) reported results were	◯ Adjusted<br>◯ Unadjusted<br>◯ Both<br>◯ Not reported/unclear
PPROM: Enter adjusted RR	__________________________________
PPROM: Was a 95% CI reported for the adjusted RR?	◯ Yes<br>◯ No
PPROM: What is the value for the lower limit for the adjusted RR?	__________________________________ (Report using two decimal places.)
PPROM: What is the value for the higher limit for the adjusted RR?	__________________________________ (Report using two decimal places.)
PPROM: Enter variables adjusted for in analysis	____ (Separate variables with a comma. If missing, enter "99999".)
PPROM: Enter unadjusted RR	__________________________________
PPROM: Was a 95% CI reported for the unadjusted RR?	◯ Yes<br>◯ No
PPROM: What is the value for the lower limit for the unadjusted RR?	__________________________________ (Report using two decimal places.)
PPROM: What is the value for the higher limit for the unadjusted RR?	__________________________________ (Report using two decimal places.)
PPROM: Was an OR reported?	◯ Yes<br>◯ No
PPROM: The (OR) reported results were	◯ Adjusted<br>◯ Unadjusted<br>◯ Both<br>◯ Not reported/unclear

PPROM: Enter adjusted OR	__________________________________
PPROM: Was a 95% CI reported for the adjusted OR?	◯ Yes<br>◯ No
PPROM: What is the value for the lower limit for the adjusted OR?	__________________________________<br>(Report using two decimal places.)
PPROM: What is the value for the higher limit for the adjusted OR?	__________________________________<br>(Report using two decimal places.)
PPROM: Enter variables adjusted for in analysis	____<br>(Separate variables with a comma. If missing, enter "99999".)
PPROM: Enter unadjusted OR	__________________________________
PPROM: Was a 95% CI reported for the unadjusted OR?	◯ Yes<br>◯ No
PPROM: What is the value for the lower limit for the unadjusted OR?	__________________________________<br>(Report using two decimal places.)
PPROM: What is the value for the higher limit for the unadjusted OR?	__________________________________<br>(Report using two decimal places.)
PPROM: Was an HR reported?	◯ Yes<br>◯ No
PPROM: The (HR) reported results were	◯ Adjusted<br>◯ Unadjusted<br>◯ Both<br>◯ Not reported/unclear
PPROM: Enter adjusted HR	__________________________________
PPROM: Was a 95% CI reported for the adjusted HR?	◯ Yes<br>◯ No
PPROM: What is the value for the lower limit for the adjusted HR?	__________________________________<br>(Report using two decimal places.)
PPROM: What is the value for the higher limit for the adjusted HR?	__________________________________<br>(Report using two decimal places.)

PPROM: Enter variables adjusted for in analysis

____
(Separate variables with a comma. If missing, enter "99999".)

PPROM: Enter unadjusted HR

____

PPROM: Was a 95% CI reported for the unadjusted HR?

◯ Yes
◯ No

PPROM: What is the value for the lower limit for the unadjusted HR?

____
(Report using two decimal places.)

PPROM: What is the value for the higher limit for the unadjusted HR?

____
(Report using two decimal places.)

Premature rupture of membranes

Premature rupture of membranes defined as spontaneous tearing of the membranes surrounding the fetus any time before the onset of obstetric labour?

◯ Yes
◯ No
◯ Not reported/unclear

Please specify definition of premature rupture of membranes

____

Number of women included in PROM analysis

____
(If missing, enter "99999".)

Were outcomes reported separately for women with diabetes?

◯ Yes
◯ No

Were outcomes reported separately for co-infections?

◯ Yes
◯ No

Were outcomes reported separately for albicans and non-albicans species?

◯ Yes
◯ No

PROM: Number with outcome and VY+

____
(If missing, enter "99999".)

PROM: Number with outcome and VY-

____
(If missing, enter "99999".)

PROM: Number without outcome and VY+

____
(If missing, enter "99999".)

PROM: Number without outcome and VY-	______________________ (If missing, enter "99999".)
Are the above numbers provided by the author or calculated from percentages?	◯ raw numbers provided<br>◯ calculated from percentage<br>◯ both
PROM: Was a RR reported?	◯ Yes<br>◯ No
PROM: The (RR) reported results were	◯ Adjusted<br>◯ Unadjusted<br>◯ Both<br>◯ Not reported/unclear
PROM: Enter adjusted RR	______________________
PROM: Was a 95% CI reported for the adjusted RR?	◯ Yes<br>◯ No
PROM: What is the value for the lower limit for the adjusted RR?	______________________ (Report using two decimal places.)
PROM: What is the value for the higher limit for the adjusted RR?	______________________ (Report using two decimal places.)
PROM: Enter variables adjusted for in analysis	______________________ (Separate variables with a comma. If missing, enter "99999".)
PROM: Enter unadjusted RR	______________________
PROM: Was a 95% CI reported for the unadjusted RR?	◯ Yes<br>◯ No
PROM: What is the value for the lower limit for the unadjusted RR?	______________________ (Report using two decimal places.)
PROM: What is the value for the higher limit for the unadjusted RR?	______________________ (Report using two decimal places.)
PROM: Was an OR reported?	◯ Yes<br>◯ No
PROM: The (OR) reported results were	◯ Adjusted<br>◯ Unadjusted<br>◯ Both<br>◯ Not reported/unclear

PROM: Enter adjusted OR	__________________________________
PROM: Was a 95% CI reported for the adjusted OR?	◯ Yes<br>◯ No
PROM: What is the value for the lower limit for the adjusted OR?	__________________________________<br>(Report using two decimal places.)
PROM: What is the value for the higher limit for the adjusted OR?	__________________________________<br>(Report using two decimal places.)
PROM: Enter variables adjusted for in analysis	____<br>(Separate variables with a comma. If missing, enter "99999".)
PROM: Enter unadjusted OR	__________________________________
PROM: Was a 95% CI reported for the unadjusted OR?	◯ Yes<br>◯ No
PROM: What is the value for the lower limit for the unadjusted OR?	__________________________________<br>(Report using two decimal places.)
PROM: What is the value for the higher limit for the unadjusted OR?	__________________________________<br>(Report using two decimal places.)
PROM: Was an HR reported?	◯ Yes<br>◯ No
PROM: The (HR) reported results were	◯ Adjusted<br>◯ Unadjusted<br>◯ Both<br>◯ Not reported/unclear
PROM: Enter adjusted HR	__________________________________
PROM: Was a 95% CI reported for the adjusted HR?	◯ Yes<br>◯ No
PROM: What is the value for the lower limit for the adjusted HR?	__________________________________<br>(Report using two decimal places.)
PROM: What is the value for the higher limit for the adjusted HR?	__________________________________<br>(Report using two decimal places.)

PROM: Enter variables adjusted for in analysis

____
(Separate variables with a comma. If missing, enter "99999".)

PROM: Enter unadjusted HR

____

PROM: Was a 95% CI reported for the unadjusted HR?
◯ Yes
◯ No

PROM: What is the value for the lower limit for the unadjusted HR?

____
(Report using two decimal places.)

PROM: What is the value for the higher limit for the unadjusted HR?

____
(Report using two decimal places.)

Low birth weight

Low birth weight defined as infant having a birth weight of less than 2500g?
◯ Yes
◯ No
◯ Not reported/unclear

Please specify definition of low birth weight

____

Number of women included in low birth weight analysis

____
(If missing, enter "99999".)

Were outcomes reported separately for women with diabetes?
◯ Yes
◯ No

Were outcomes reported separately for co-infections?
◯ Yes
◯ No

Were outcomes reported separately for albicans and non-albicans species?
◯ Yes
◯ No

Low birth weight: Number with outcome and VY+

____
(If missing, enter "99999".)

Low birth weight: Number with outcome and VY-

____
(If missing, enter "99999".)

Low birth weight: Number without outcome and VY+

____
(If missing, enter "99999".)

Low birth weight: Number without outcome and VY-	__________________ (If missing, enter "99999".)
Are the above numbers provided by the author or calculated from percentages?	◯ raw numbers provided<br>◯ calculated from percentage<br>◯ both
Low birth weight: Was a RR reported?	◯ Yes<br>◯ No
Low birth weight: The (RR) reported results were	◯ Adjusted<br>◯ Unadjusted<br>◯ Both<br>◯ Not reported/unclear
Low birth weight: Enter adjusted RR	__________________
Low birth weight: Was a 95% CI reported for the adjusted RR?	◯ Yes<br>◯ No
Low birth weight: What is the value for the lower limit for the adjusted RR?	__________________ (Report using two decimal places.)
Low birth weight: What is the value for the higher limit for the adjusted RR?	__________________ (Report using two decimal places.)
Low birth weight: Enter variables adjusted for in analysis	__________________ (Separate variables with a comma. If missing, enter "99999".)
Low birth weight: Enter unadjusted RR	__________________
Low birth weight: Was a 95% CI reported for the unadjusted RR?	◯ Yes<br>◯ No
Low birth weight: What is the value for the lower limit for the unadjusted RR?	__________________ (Report using two decimal places.)
Low birth weight: What is the value for the higher limit for the unadjusted RR?	__________________ (Report using two decimal places.)
Low birth weight: Was an OR reported?	◯ Yes<br>◯ No
Low birth weight: The (OR) reported results were	◯ Adjusted<br>◯ Unadjusted<br>◯ Both<br>◯ Not reported/unclear

Low birth weight: Enter adjusted OR	__________________________________
Low birth weight: Was a 95% CI reported for the adjusted OR?	◯ Yes<br>◯ No
Low birth weight: What is the value for the lower limit for the adjusted OR?	__________________________________<br>(Report using two decimal places.)
Low birth weight: What is the value for the higher limit for the adjusted OR?	__________________________________<br>(Report using two decimal places.)
Low birth weight: Enter variables adjusted for in analysis	____<br>(Separate variables with a comma. If missing, enter "99999".)
Low birth weight: Enter unadjusted OR	__________________________________
Low birth weight: Was a 95% CI reported for the unadjusted OR?	◯ Yes<br>◯ No
Low birth weight: What is the value for the lower limit for the unadjusted OR?	__________________________________<br>(Report using two decimal places.)
Low birth weight: What is the value for the higher limit for the unadjusted OR?	__________________________________<br>(Report using two decimal places.)
Low birth weight: Was an HR reported?	◯ Yes<br>◯ No
Low birth weight: The (HR) reported results were	◯ Adjusted<br>◯ Unadjusted<br>◯ Both<br>◯ Not reported/unclear
Low birth weight: Enter adjusted HR	__________________________________
Low birth weight: Was a 95% CI reported for the adjusted HR?	◯ Yes<br>◯ No
Low birth weight: What is the value for the lower limit for the adjusted HR?	__________________________________<br>(Report using two decimal places.)
Low birth weight: What is the value for the higher limit for the adjusted HR?	__________________________________<br>(Report using two decimal places.)

Low birth weight: Enter variables adjusted for in analysis	______________________ (Separate variables with a comma. If missing, enter "99999".)
Low birth weight: Enter unadjusted HR	______________________
Low birth weight: Was a 95% CI reported for the unadjusted HR?	◯ Yes ◯ No
Low birth weight: What is the value for the lower limit for the unadjusted HR?	______________________ (Report using two decimal places.)
Low birth weight: What is the value for the higher limit for the unadjusted HR?	______________________ (Report using two decimal places.)

Small for getational age

Small for gestational age defined as infant having a birth weight below 10th centile for gestational age?	◯ Yes ◯ No ◯ Not reported/unclear
Please specify definition of small for gestational age	______________________
Number of women included in small for gestational age analysis	______________________ (If missing, enter "99999".)
Were outcomes reported separately for women with diabetes?	◯ Yes ◯ No
Were outcomes reported separately for co-infections?	◯ Yes ◯ No
Were outcomes reported separately for albicans and non-albicans species?	◯ Yes ◯ No
Small for gestational age: Number with outcome and VY+	______________________ (If missing, enter "99999".)
Small for gestational age: Number with outcome and VY-	______________________ (If missing, enter "99999".)
Small for gestational age: Number without outcome and VY+	______________________ (If missing, enter "99999".)

Small for gestational age: Number without outcome and VY-	______________________ (If missing, enter "99999".)
Are the above numbers provided by the author or calculated from percentages?	◯ raw numbers provided<br>◯ calculated from percentage<br>◯ both
Small for gestational age: Was a RR reported?	◯ Yes<br>◯ No
Small for gestational age: The (RR) reported results were	◯ Adjusted<br>◯ Unadjusted<br>◯ Both<br>◯ Not reported/unclear
Small for gestational age: Enter adjusted RR	______________________
Small for gestational age: Was a 95% CI reported for the adjusted RR?	◯ Yes<br>◯ No
Small for gestational age: What is the value for the lower limit for the adjusted RR?	______________________ (Report using two decimal places.)
Small for gestational age: What is the value for the higher limit for the adjusted RR?	______________________ (Report using two decimal places.)
Small for gestational age: Enter variables adjusted for in analysis	______________________ (Separate variables with a comma. If missing, enter "99999".)
Small for gestational age: Enter unadjusted RR	______________________
Small for gestational age: Was a 95% CI reported for the unadjusted RR?	◯ Yes<br>◯ No
Small for gestational age: What is the value for the lower limit for the unadjusted RR?	______________________ (Report using two decimal places.)
Small for gestational age: What is the value for the higher limit for the unadjusted RR?	______________________ (Report using two decimal places.)
Small for gestational age: Was an OR reported?	◯ Yes<br>◯ No
Small for gestational age: The (OR) reported results were	◯ Adjusted<br>◯ Unadjusted<br>◯ Both<br>◯ Not reported/unclear

Small for gestational age: Enter adjusted OR	______________________
Small for gestational age: Was a 95% CI reported for the adjusted OR?	◯ Yes<br>◯ No
Small for gestational age: What is the value for the lower limit for the adjusted OR?	______________________<br>(Report using two decimal places.)
Small for gestational age: What is the value for the higher limit for the adjusted OR?	______________________<br>(Report using two decimal places.)
Small for gestational age: Enter variables adjusted for in analysis	______________________<br>(Separate variables with a comma. If missing, enter "99999".)
Small for gestational age: Enter unadjusted OR	______________________
Small for gestational age: Was a 95% CI reported for the unadjusted OR?	◯ Yes<br>◯ No
Small for gestational age: What is the value for the lower limit for the unadjusted OR?	______________________<br>(Report using two decimal places.)
Small for gestational age: What is the value for the higher limit for the unadjusted OR?	______________________<br>(Report using two decimal places.)
Small for gestational age: Was an HR reported?	◯ Yes<br>◯ No
Small for gestational age: The (HR) reported results were	◯ Adjusted<br>◯ Unadjusted<br>◯ Both<br>◯ Not reported/unclear
Small for gestational age: Enter adjusted HR	______________________
Small for gestational age: Was a 95% CI reported for the adjusted HR?	◯ Yes<br>◯ No
Small for gestational age: What is the value for the lower limit for the adjusted HR?	______________________<br>(Report using two decimal places.)
Small for gestational age: What is the value for the higher limit for the adjusted HR?	______________________<br>(Report using two decimal places.)

Small for gestational age: Enter variables adjusted for in analysis

____
(Separate variables with a comma. If missing, enter "99999".)

Small for gestational age: Enter unadjusted HR

____

Small for gestational age: Was a 95% CI reported for the unadjusted HR?

- ◯ Yes
- ◯ No

Small for gestational age: What is the value for the lower limit for the unadjusted HR?

____
(Report using two decimal places.)

Small for gestational age: What is the value for the higher limit for the unadjusted HR?

____
(Report using two decimal places.)

Inflammation of the placenta or uterus

Inflammation of the placenta or uterus defined as inflammation of the endometrium, the placental membranes, and connected tissues such as fetal blood vessels and umbilical cord?

- ◯ Yes
- ◯ No
- ◯ Not reported/unclear

Please specify definition of inflammation of the placenta or uterus

____

Please specify type of inflammation

- ☐ Chorioamnionitis
- ☐ Endometritis
- ☐ Funisitis
- ☐ Villitis
- ☐ Ohter/unspecified

Number of women included in inflammation of the placenta or uterus analysis

____
(If missing, enter "99999".)

Were outcomes reported separately for women with diabetes?

- ◯ Yes
- ◯ No

Were outcomes reported separately for co-infections?

- ◯ Yes
- ◯ No

Were outcomes reported separately for albicans and non-albicans species?

- ◯ Yes
- ◯ No

Inflammation of the placenta or uterus: Number with outcome and VY+

____
(If missing, enter "99999".)

Inflammation of the placenta or uterus: Number with outcome and VY-

____
(If missing, enter "99999".)

Inflammation of the placenta or uterus: Number without outcome and VY+	________________ (If missing, enter "99999".)
Inflammation of the placenta or uterus: Number without outcome and VY-	________________ (If missing, enter "99999".)
Are the above numbers provided by the author or calculated from percentages?	◯ raw numbers provided<br>◯ calculated from percentage<br>◯ both
Inflammation of the placenta or uterus: Was a RR reported?	◯ Yes<br>◯ No
Inflammation of the placenta or uterus: The (RR) reported results were	◯ Adjusted<br>◯ Unadjusted<br>◯ Both<br>◯ Not reported/unclear
Inflammation of the placenta or uterus: Enter adjusted RR	________________
Inflammation of the placenta or uterus: Was a 95% CI reported for the adjusted RR?	◯ Yes<br>◯ No
Inflammation of the placenta or uterus: What is the value for the lower limit for the adjusted RR?	________________ (Report using two decimal places.)
Inflammation of the placenta or uterus: What is the value for the higher limit for the adjusted RR?	________________ (Report using two decimal places.)
Inflammation of the placenta or uterus: Enter variables adjusted for in analysis	________________ (Separate variables with a comma. If missing, enter "99999".)
Inflammation of the placenta or uterus: Enter unadjusted RR	________________
Inflammation of the placenta or uterus: Was a 95% CI reported for the unadjusted RR?	◯ Yes<br>◯ No
Inflammation of the placenta or uterus: What is the value for the lower limit for the unadjusted RR?	________________ (Report using two decimal places.)
Inflammation of the placenta or uterus: What is the value for the higher limit for the unadjusted RR?	________________ (Report using two decimal places.)
Inflammation of the placenta or uterus: Was an OR reported?	◯ Yes<br>◯ No

Inflammation of the placenta or uterus: The (OR) reported results were	◯ Adjusted<br>◯ Unadjusted<br>◯ Both<br>◯ Not reported/unclear
Inflammation of the placenta or uterus: Enter adjusted OR	__________________________________
Inflammation of the placenta or uterus: Was a 95% CI reported for the adjusted OR?	◯ Yes<br>◯ No
Inflammation of the placenta or uterus: What is the value for the lower limit for the adjusted OR?	__________________________________<br>(Report using two decimal places.)
Inflammation of the placenta or uterus: What is the value for the higher limit for the adjusted OR?	__________________________________<br>(Report using two decimal places.)
Inflammation of the placenta or uterus: Enter variables adjusted for in analysis	____<br>(Separate variables with a comma. If missing, enter "99999".)
Inflammation of the placenta or uterus: Enter unadjusted OR	__________________________________
Inflammation of the placenta or uterus: Was a 95% CI reported for the unadjusted OR?	◯ Yes<br>◯ No
Inflammation of the placenta or uterus: What is the value for the lower limit for the unadjusted OR?	__________________________________<br>(Report using two decimal places.)
Inflammation of the placenta or uterus: What is the value for the higher limit for the unadjusted OR?	__________________________________<br>(Report using two decimal places.)
Inflammation of the placenta or uterus: Was an HR reported?	◯ Yes<br>◯ No
Inflammation of the placenta or uterus: The (HR) reported results were	◯ Adjusted<br>◯ Unadjusted<br>◯ Both<br>◯ Not reported/unclear
Inflammation of the placenta or uterus: Enter adjusted HR	__________________________________
Inflammation of the placenta or uterus: Was a 95% CI reported for the adjusted HR?	◯ Yes<br>◯ No
Inflammation of the placenta or uterus: What is the value for the lower limit for the adjusted HR?	__________________________________<br>(Report using two decimal places.)

Inflammation of the placenta or uterus: What is the value for the higher limit for the adjusted HR?	______________________ (Report using two decimal places.)
Inflammation of the placenta or uterus: Enter variables adjusted for in analysis	______________________ (Separate variables with a comma. If missing, enter "99999".)
Inflammation of the placenta or uterus: Enter unadjusted HR	______________________
Inflammation of the placenta or uterus: Was a 95% CI reported for the unadjusted HR?	◯ Yes<br>◯ No
Inflammation of the placenta or uterus: What is the value for the lower limit for the unadjusted HR?	______________________ (Report using two decimal places.)
Inflammation of the placenta or uterus: What is the value for the higher limit for the unadjusted HR?	______________________ (Report using two decimal places.)

Neonatal death

Neonatal death defined as death of a live-born infant during the first 28 completed days of life?	◯ Yes<br>◯ No<br>◯ Not reported/unclear
Please specify definition of neonatal death	______________________
Number of women included in neonatal death analysis	______________________ (If missing, enter "99999".)
Were outcomes reported separately for women with diabetes?	◯ Yes<br>◯ No
Were outcomes reported separately for co-infections?	◯ Yes<br>◯ No
Were outcomes reported separately for albicans and non-albicans species?	◯ Yes<br>◯ No
Neonatal death: Number with outcome and VY+	______________________ (If missing, enter "99999".)
Neonatal death: Number with outcome and VY-	______________________ (If missing, enter "99999".)

Neonatal death: Number without outcome and VY+	__________________________________ (If missing, enter "99999".)
Neonatal death: Number without outcome and VY-	__________________________________ (If missing, enter "99999".)
Are the above numbers provided by the author or calculated from percentages?	◯ raw numbers provided<br>◯ calculated from percentage<br>◯ both
Neonatal death: Was a RR reported?	◯ Yes<br>◯ No
Neonatal death: The (RR) reported results were	◯ Adjusted<br>◯ Unadjusted<br>◯ Both<br>◯ Not reported/unclear
Neonatal death: Enter adjusted RR	__________________________________
Neonatal death: Was a 95% CI reported for the adjusted RR?	◯ Yes<br>◯ No
Neonatal death: What is the value for the lower limit for the adjusted RR?	__________________________________ (Report using two decimal places.)
Neonatal death: What is the value for the higher limit for the adjusted RR?	__________________________________ (Report using two decimal places.)
Neonatal death: Enter variables adjusted for in analysis	____ (Separate variables with a comma. If missing, enter "99999".)
Neonatal death: Enter unadjusted RR	__________________________________
Neonatal death: Was a 95% CI reported for the unadjusted RR?	◯ Yes<br>◯ No
Neonatal death: What is the value for the lower limit for the unadjusted RR?	__________________________________ (Report using two decimal places.)
Neonatal death: What is the value for the higher limit for the unadjusted RR?	__________________________________ (Report using two decimal places.)
Neonatal death: Was an OR reported?	◯ Yes<br>◯ No

Neonatal death: The (OR) reported results were	◯ Adjusted<br>◯ Unadjusted<br>◯ Both<br>◯ Not reported/unclear
Neonatal death: Enter adjusted OR	__________________________________
Neonatal death: Was a 95% CI reported for the adjusted OR?	◯ Yes<br>◯ No
Neonatal death: What is the value for the lower limit for the adjusted OR?	__________________________________<br>(Report using two decimal places.)
Neonatal death: What is the value for the higher limit for the adjusted OR?	__________________________________<br>(Report using two decimal places.)
Neonatal death: Enter variables adjusted for in analysis	__________________________________<br>(Separate variables with a comma. If missing, enter "99999".)
Neonatal death: Enter unadjusted OR	__________________________________
Neonatal death: Was a 95% CI reported for the unadjusted OR?	◯ Yes<br>◯ No
Neonatal death: What is the value for the lower limit for the unadjusted OR?	__________________________________<br>(Report using two decimal places.)
Neonatal death: What is the value for the higher limit for the unadjusted OR?	__________________________________<br>(Report using two decimal places.)
Neonatal death: Was an HR reported?	◯ Yes<br>◯ No
Neonatal death: The (HR) reported results were	◯ Adjusted<br>◯ Unadjusted<br>◯ Both<br>◯ Not reported/unclear
Neonatal death: Enter adjusted HR	__________________________________
Neonatal death: Was a 95% CI reported for the adjusted HR?	◯ Yes<br>◯ No
Neonatal death: What is the value for the lower limit for the adjusted HR?	__________________________________<br>(Report using two decimal places.)

Neonatal death: What is the value for the higher limit for the adjusted HR?

____
(Report using two decimal places.)

Neonatal death: Enter variables adjusted for in analysis

____
(Separate variables with a comma. If missing, enter "99999".)

Neonatal death: Enter unadjusted HR

____

Neonatal death: Was a 95% CI reported for the unadjusted HR?

◯ Yes
◯ No

Neonatal death: What is the value for the lower limit for the unadjusted HR?

____
(Report using two decimal places.)

Neonatal death: What is the value for the higher limit for the unadjusted HR?

____
(Report using two decimal places.)

Neonatal fungal sepsis

Neonatal fungal sepsis defined as culture confirmed fungemia?

◯ Yes
◯ No
◯ Not reported/unclear

Please specify definition of neonatal fungal sepsis

____

Number of women included in neonatal fungal sepsis analysis

____
(If missing, enter "99999".)

Were outcomes reported separately for women with diabetes?

◯ Yes
◯ No

Were outcomes reported separately for co-infections?

◯ Yes
◯ No

Were outcomes reported separately for albicans and non-albicans species?

◯ Yes
◯ No

Neonatal fungal sepsis: Number with outcome and VY+

____
(If missing, enter "99999".)

Neonatal fungal sepsis: Number with outcome and VY-

____
(If missing, enter "99999".)

Neonatal fungal sepsis: Number without outcome and VY+	__________________________________<br>(If missing, enter "99999".)
Neonatal fungal sepsis: Number without outcome and VY-	__________________________________<br>(If missing, enter "99999".)
Are the above numbers provided by the author or calculated from percentages?	◯ raw numbers provided<br>◯ calculated from percentage<br>◯ both
Neonatal fungal sepsis: Was a RR reported?	◯ Yes<br>◯ No
Neonatal fungal sepsis: The (RR) reported results were	◯ Adjusted<br>◯ Unadjusted<br>◯ Both<br>◯ Not reported/unclear
Neonatal fungal sepsis: Enter adjusted RR	__________________________________
Neonatal fungal sepsis: Was a 95% CI reported for the adjusted RR?	◯ Yes<br>◯ No
Neonatal fungal sepsis: What is the value for the lower limit for the adjusted RR?	__________________________________<br>(Report using two decimal places.)
Neonatal fungal sepsis: What is the value for the higher limit for the adjusted RR?	__________________________________<br>(Report using two decimal places.)
Neonatal fungal sepsis: Enter variables adjusted for in analysis	____<br>(Separate variables with a comma. If missing, enter "99999".)
Neonatal fungal sepsis: Enter unadjusted RR	__________________________________
Neonatal fungal sepsis: Was a 95% CI reported for the unadjusted RR?	◯ Yes<br>◯ No
Neonatal fungal sepsis: What is the value for the lower limit for the unadjusted RR?	__________________________________<br>(Report using two decimal places.)
Neonatal fungal sepsis: What is the value for the higher limit for the unadjusted RR?	__________________________________<br>(Report using two decimal places.)
Neonatal fungal sepsis: Was an OR reported?	◯ Yes<br>◯ No

Neonatal fungal sepsis: The (OR) reported results were	◯ Adjusted<br>◯ Unadjusted<br>◯ Both<br>◯ Not reported/unclear
Neonatal fungal sepsis: Enter adjusted OR	__________________________________
Neonatal fungal sepsis: Was a 95% CI reported for the adjusted OR?	◯ Yes<br>◯ No
Neonatal fungal sepsis: What is the value for the lower limit for the adjusted OR?	__________________________________<br>(Report using two decimal places.)
Neonatal fungal sepsis: What is the value for the higher limit for the adjusted OR?	__________________________________<br>(Report using two decimal places.)
Neonatal fungal sepsis: Enter variables adjusted for in analysis	__________________________________<br>(Separate variables with a comma. If missing, enter "99999".)
Neonatal fungal sepsis: Enter unadjusted OR	__________________________________
Neonatal fungal sepsis: Was a 95% CI reported for the unadjusted OR?	◯ Yes<br>◯ No
Neonatal fungal sepsis: What is the value for the lower limit for the unadjusted OR?	__________________________________<br>(Report using two decimal places.)
Neonatal fungal sepsis: What is the value for the higher limit for the unadjusted OR?	__________________________________<br>(Report using two decimal places.)
Neonatal fungal sepsis: Was an HR reported?	◯ Yes<br>◯ No
Neonatal fungal sepsis: The (HR) reported results were	◯ Adjusted<br>◯ Unadjusted<br>◯ Both<br>◯ Not reported/unclear
Neonatal fungal sepsis: Enter adjusted HR	__________________________________
Neonatal fungal sepsis: Was a 95% CI reported for the adjusted HR?	◯ Yes<br>◯ No
Neonatal fungal sepsis: What is the value for the lower limit for the adjusted HR?	__________________________________<br>(Report using two decimal places.)

Neonatal fungal sepsis: What is the value for the higher limit for the adjusted HR?

____
(Report using two decimal places.)

Neonatal fungal sepsis: Enter variables adjusted for in analysis

____
(Separate variables with a comma. If missing, enter "99999".)

Neonatal fungal sepsis: Enter unadjusted HR

____

Neonatal fungal sepsis: Was a 95% CI reported for the unadjusted HR?

◯ Yes
◯ No

Neonatal fungal sepsis: What is the value for the lower limit for the unadjusted HR?

____
(Report using two decimal places.)

Neonatal fungal sepsis: What is the value for the higher limit for the unadjusted HR?

____
(Report using two decimal places.)

Comments

Please enter any additional comments:

____

Extracted by?

____
(Enter initials only.)

Verified by?

____
(Enter initials only.)

Risk of Bias assessment (person 1)

Cohort study

Was assessing this association an objective?	◯ Yes, primary objective<br>◯ Yes, other objective<br>◯ No
Comments:	__________________________________
Was selection of exposed and non-exposed cohorts drawn from the same population?	◯ Definitely yes (low risk of bias)<br>◯ Probably yes<br>◯ Probably no<br>◯ Definitely no (high risk of bias)
Comments:	__________________________________
Can we be confident in the assessment of exposure?	◯ Definitely yes (low risk of bias)<br>◯ Probably yes<br>◯ Probably no<br>◯ Definitely no (high risk of bias)
Comments:	__________________________________
Can we be confident that the outcome of interest was not present at start of study?	◯ Definitely yes (low risk of bias)<br>◯ Probably yes<br>◯ Probably no<br>◯ Definitely no (high risk of bias)
Comments:	__________________________________
Did the statistical analysis adjust for confounding factors?	◯ Definitely yes (low risk of bias)<br>◯ Probably yes<br>◯ Probably no<br>◯ Definitely no (high risk of bias)
Comments:	__________________________________
Can we be confident in the assessment of the presence or absence of prognostic factors?	◯ Definitely yes (low risk of bias)<br>◯ Probably yes<br>◯ Probably no<br>◯ Definitely no (high risk of bias)
Comments:	__________________________________

Can we be confident in the assessment of outcome?	◯ Definitely yes (low risk of bias)<br>◯ Probably yes<br>◯ Probably no<br>◯ Definitely no (high risk of bias)
Comments:	__________________________________
Was the follow up of cohorts adequate?	◯ Definitely yes (low risk of bias)<br>◯ Probably yes<br>◯ Probably no<br>◯ Definitely no (high risk of bias)
Comments:	__________________________________
Were co-interventions similar between groups?	◯ Definitely yes (low risk of bias)<br>◯ Probably yes<br>◯ Probably no<br>◯ Definitely no (high risk of bias)
Comments:	__________________________________
Extracted by?	____________________________<br>(Enter initials only.)
Case-control study	
Was assessing this association an objective?	◯ Yes, primary objective<br>◯ Yes, other objective<br>◯ No
Comments:	__________________________________
Can we be confident in the assessment of exposure?	◯ Definitely yes (low risk of bias)<br>◯ Probably yes<br>◯ Probably no<br>◯ Definitely no (high risk of bias)
Comments:	__________________________________
Can we be confident that cases had developed the outcome of interest and controls had not?	◯ Definitely yes (low risk of bias)<br>◯ Probably yes<br>◯ Probably no<br>◯ Definitely no (high risk of bias)

Comments:

____

Were the cases (those who were exposed and developed the outcome of interest) properly selected?	◯ Definitely yes (low risk of bias)<br>◯ Probably yes<br>◯ Probably no<br>◯ Definitely no (high risk of bias)

Comments:

____

Were the controls (those who were exposed and did not develop the outcome of interest) properly selected?	◯ Definitely yes (low risk of bias)<br>◯ Probably yes<br>◯ Probably no<br>◯ Definitely no (high risk of bias)

Comments:

____

Were cases and controls matched according to important prognostic variables or was statistical adjustment carried out for those variables?	◯ Definitely yes (low risk of bias)<br>◯ Probably yes<br>◯ Probably no<br>◯ Definitely no (high risk of bias)

Comments:

____

Extracted by?

____
(Enter initials only.)

Risk of Bias assessment (person 2)

Cohort study	
Was assessing this association an objective?	◯ Yes, primary objective<br>◯ Yes, other objective<br>◯ No
Comments:	__________________
Was selection of exposed and non-exposed cohorts drawn from the same population?	◯ Definitely yes (low risk of bias)<br>◯ Probably yes<br>◯ Probably no<br>◯ Definitely no (high risk of bias)
Comments:	__________________
Can we be confident in the assessment of exposure?	◯ Definitely yes (low risk of bias)<br>◯ Probably yes<br>◯ Probably no<br>◯ Definitely no (high risk of bias)
Comments:	__________________
Can we be confident that the outcome of interest was not present at start of study?	◯ Definitely yes (low risk of bias)<br>◯ Probably yes<br>◯ Probably no<br>◯ Definitely no (high risk of bias)
Comments:	__________________
Did the statistical analysis adjust for confounding factors?	◯ Definitely yes (low risk of bias)<br>◯ Probably yes<br>◯ Probably no<br>◯ Definitely no (high risk of bias)
Comments:	__________________
Can we be confident in the assessment of the presence or absence of prognostic factors?	◯ Definitely yes (low risk of bias)<br>◯ Probably yes<br>◯ Probably no<br>◯ Definitely no (high risk of bias)
Comments:	__________________

Can we be confident in the assessment of outcome?	◯ Definitely yes (low risk of bias)<br>◯ Probably yes<br>◯ Probably no<br>◯ Definitely no (high risk of bias)
Comments:	__________________________________
Was the follow up of cohorts adequate?	◯ Definitely yes (low risk of bias)<br>◯ Probably yes<br>◯ Probably no<br>◯ Definitely no (high risk of bias)
Comments:	__________________________________
Were co-interventions similar between groups?	◯ Definitely yes (low risk of bias)<br>◯ Probably yes<br>◯ Probably no<br>◯ Definitely no (high risk of bias)
Comments:	__________________________________
Extracted by?	___________________________<br>(Enter initials only.)
Case-control study	
Was assessing this association an objective?	◯ Yes, primary objective<br>◯ Yes, other objective<br>◯ No
Comments:	__________________________________
Can we be confident in the assessment of exposure?	◯ Definitely yes (low risk of bias)<br>◯ Probably yes<br>◯ Probably no<br>◯ Definitely no (high risk of bias)
Comments:	__________________________________
Can we be confident that cases had developed the outcome of interest and controls had not?	◯ Definitely yes (low risk of bias)<br>◯ Probably yes<br>◯ Probably no<br>◯ Definitely no (high risk of bias)

Comments:

____

Were the cases (those who were exposed and developed the outcome of interest) properly selected?

- ◯ Definitely yes (low risk of bias)
- ◯ Probably yes
- ◯ Probably no
- ◯ Definitely no (high risk of bias)

Comments:

____

Were the controls (those who were exposed and did not develop the outcome of interest) properly selected?

- ◯ Definitely yes (low risk of bias)
- ◯ Probably yes
- ◯ Probably no
- ◯ Definitely no (high risk of bias)

Comments:

____

Were cases and controls matched according to important prognostic variables or was statistical adjustment carried out for those variables?

- ◯ Definitely yes (low risk of bias)
- ◯ Probably yes
- ◯ Probably no
- ◯ Definitely no (high risk of bias)

Comments:

____

Extracted by?

____
(Enter initials only.)
