# Supplementary material for: Vulvovaginal yeast infections during pregnancy and perinatal outcomes: systematic review and meta-analysis
Source: BMC Womens Health. 2023 Mar 21;23:116. doi: 10.1186/s12905-023-02258-7 (PMC10029297; doi:10.1186/s12905-023-02258-7)
Supplement: Supplementary file 3 — Additional file 3. Forest plots of stratified meta-analyses. Forest plots of meta-analyses about vulvovaginal yeast infection and preterm birth stratified by study design, diagnostic method used, income setting, and time of testing. [file 12905_2023_2258_MOESM3_ESM.docx]

**Additional file 3 - Forest plots of stratified meta-analyses**

Preterm birth stratified by study design


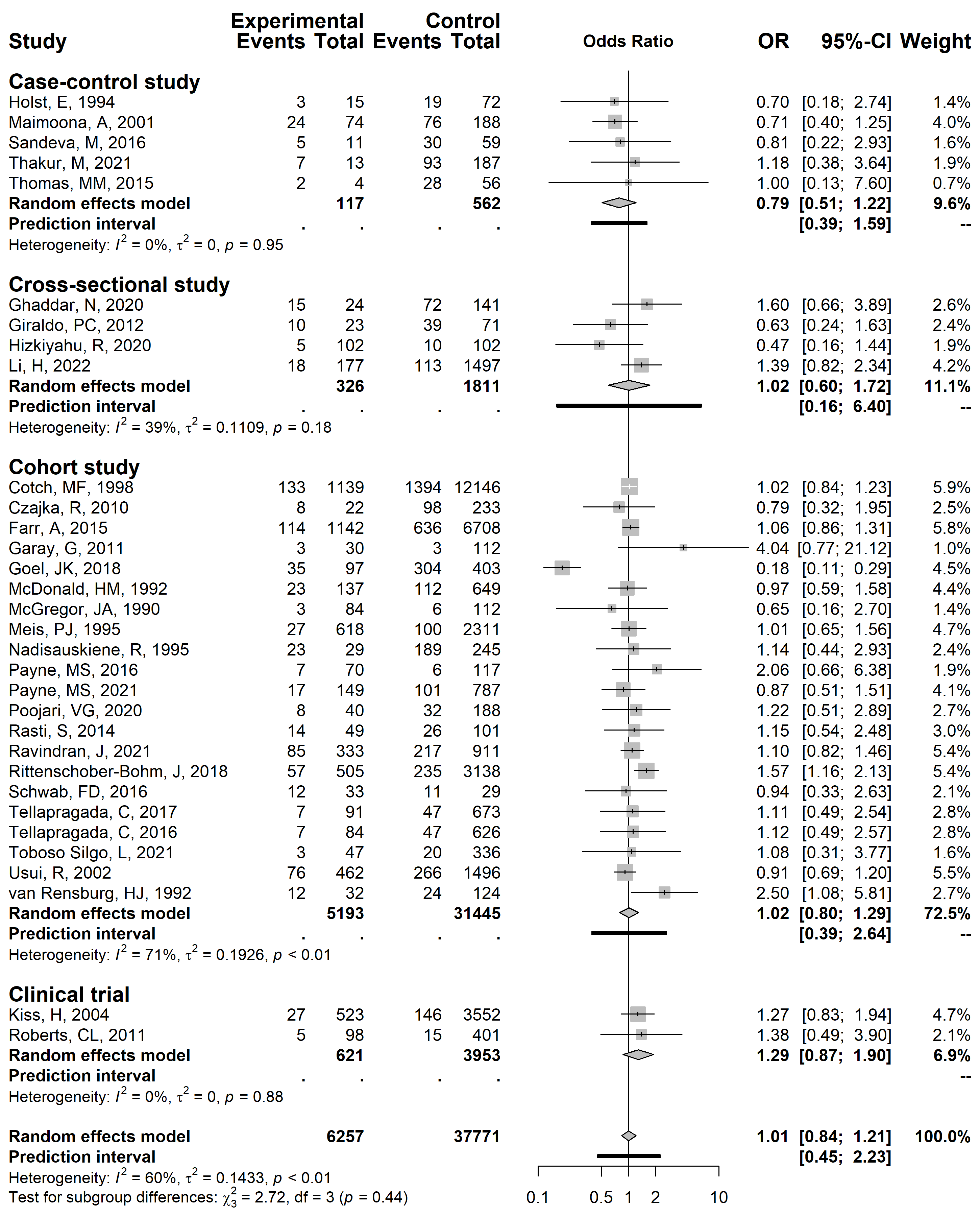


Legend: vertical line, line of no association (odds ratio 1.0); horizontal line, 95% confidence interval; vertical line inside the box, point estimate of odds ratio; grey box, study size; diamond, summary estimate with 95% confidence interval; black bar, 95% prediction interval. To the left of the line of no association, preterm birth was less likely in women with vulvovaginal yeast infection; to the right of the line of no association, preterm birth was more likely.

Preterm birth stratified by diagnostic method used


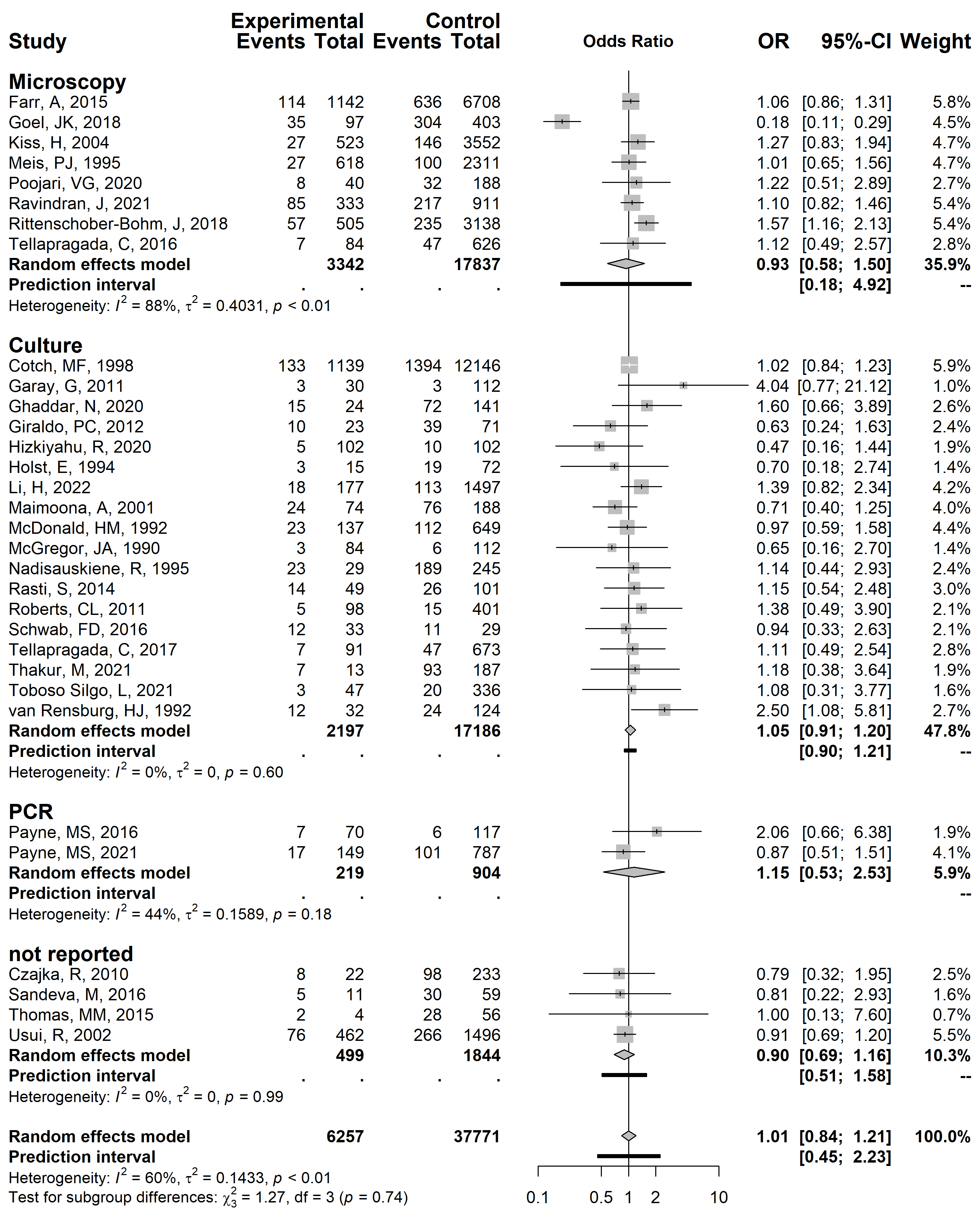


Legend: vertical line, line of no association (odds ratio 1.0); horizontal line, 95% confidence interval; vertical line inside the box, point estimate of odds ratio; grey box, study size; diamond, summary estimate with 95% confidence interval; black bar, 95% prediction interval. To the left of the line of no association, preterm birth was less likely in women with vulvovaginal yeast infection; to the right of the line of no association, preterm birth was more likely.

Preterm birth stratified by income setting


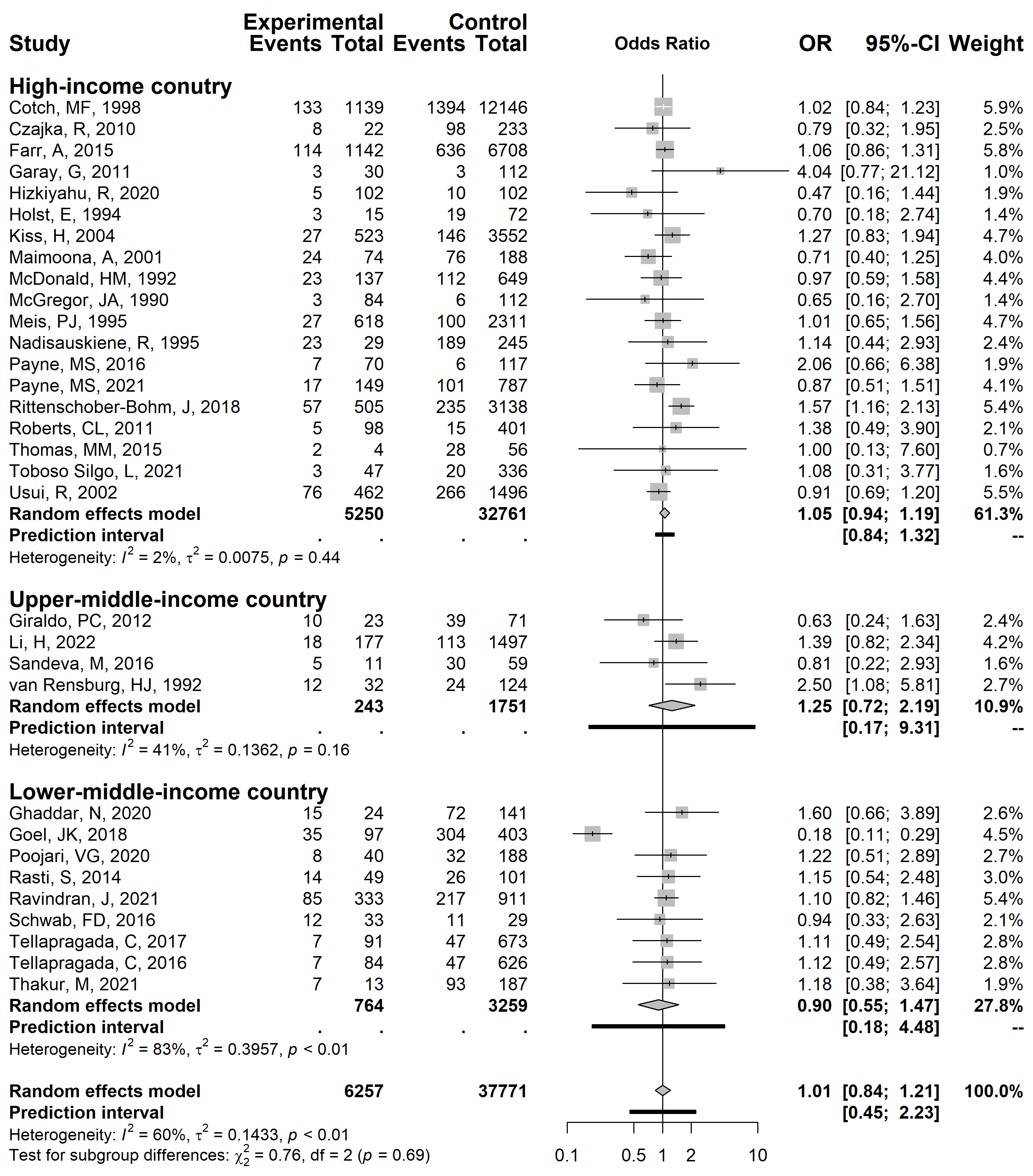


Legend: vertical line, line of no association (odds ratio 1.0); horizontal line, 95% confidence interval; vertical line inside the box, point estimate of odds ratio; grey box, study size; diamond, summary estimate with 95% confidence interval; black bar, 95% prediction interval. To the left of the line of no association, preterm birth was less likely in women with vulvovaginal yeast infection; to the right of the line of no association, preterm birth was more likely.

Preterm birth stratified by time of testing


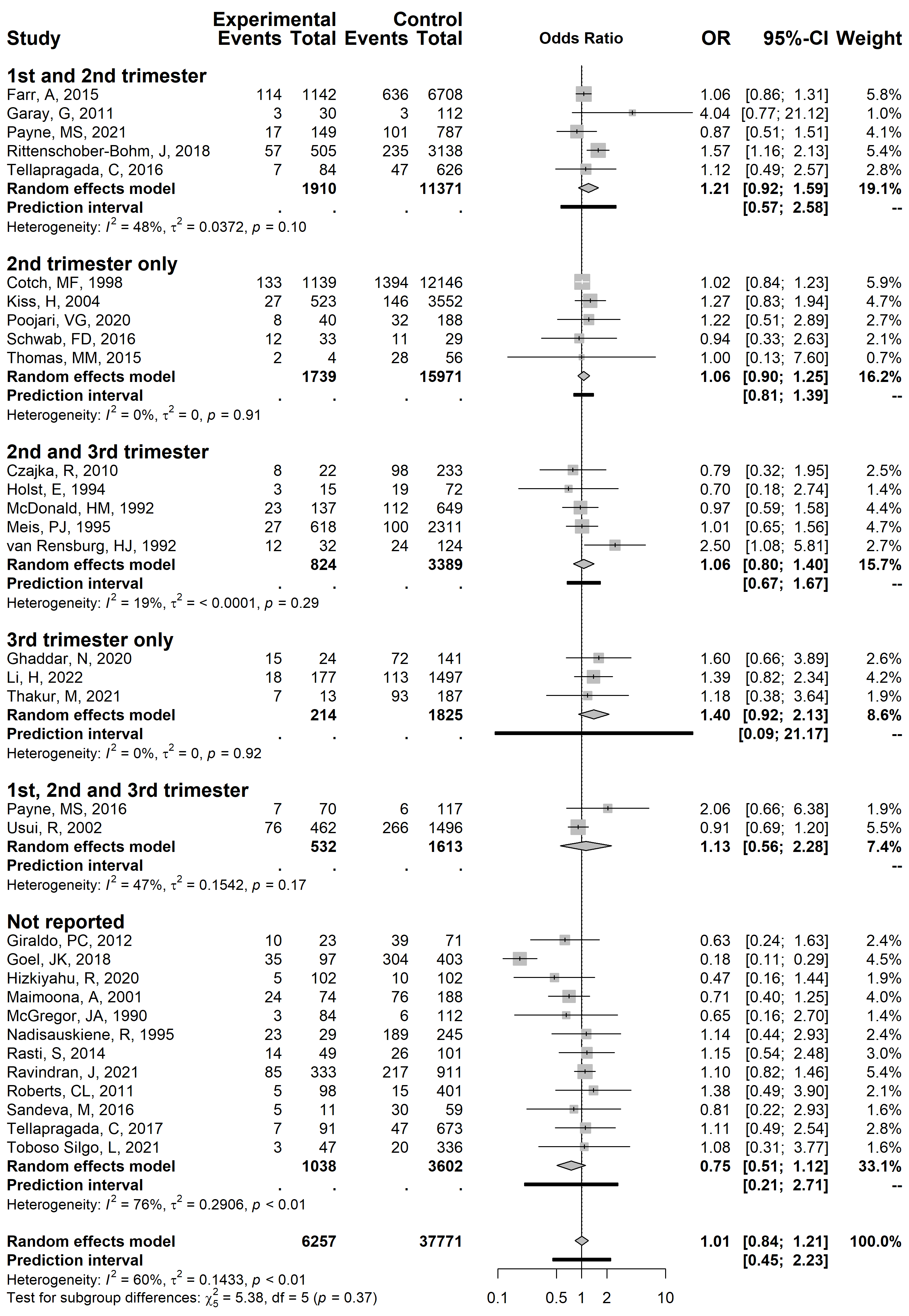


Legend: vertical line, line of no association (odds ratio 1.0); horizontal line, 95% confidence interval; vertical line inside the box, point estimate of odds ratio; grey box, study size; diamond, summary estimate with 95% confidence interval; black bar, 95% prediction interval. To the left of the line of no association, preterm birth was less likely in women with vulvovaginal yeast infection; to the right of the line of no association, preterm birth was more likely.
