# Supplementary material for: Vulvovaginal yeast infections during pregnancy and perinatal outcomes: systematic review and meta-analysis
Source: BMC Womens Health. 2023 Mar 21;23:116. doi: 10.1186/s12905-023-02258-7 (PMC10029297; doi:10.1186/s12905-023-02258-7)
Supplement: Supplementary file 4 — Additional file 4. Forest plots of secondary outcomes. Forest plots of meta-analyses about vulvovaginal yeast infection and spontaneous abortion, stillbirth, preterm premature rupture of membranes, premature rupture of membranes, low birth weight, inflammation of the placenta or uterus. [file 12905_2023_2258_MOESM4_ESM.docx]

**Additional file 4 - Forest plots of secondary outcomes**

Spontaneous abortion


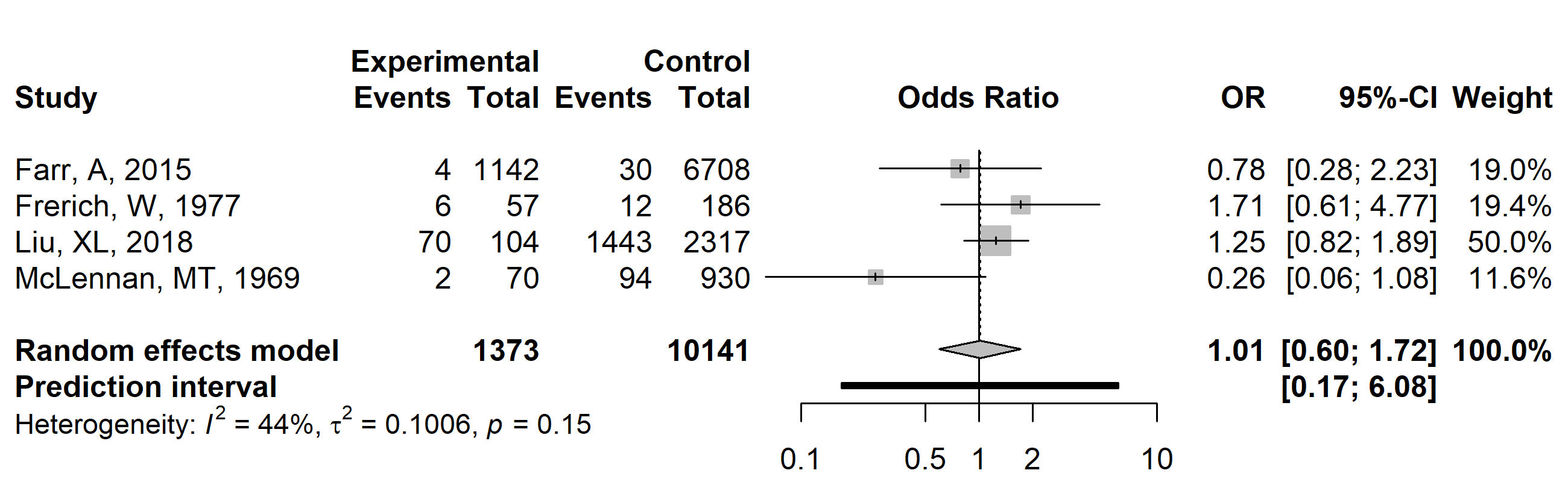


Legend: vertical line, line of no association (odds ratio 1.0); horizontal line, 95% confidence interval; vertical line inside the box, point estimate of odds ratio; grey box, study size; diamond, summary estimate with 95% confidence interval; black bar, 95% prediction interval. To the left of the line of no association, spontaneous abortion was less likely in women with vulvovaginal yeast infection; to the right of the line of no association, spontaneous abortion was more likely.

Stillbirth


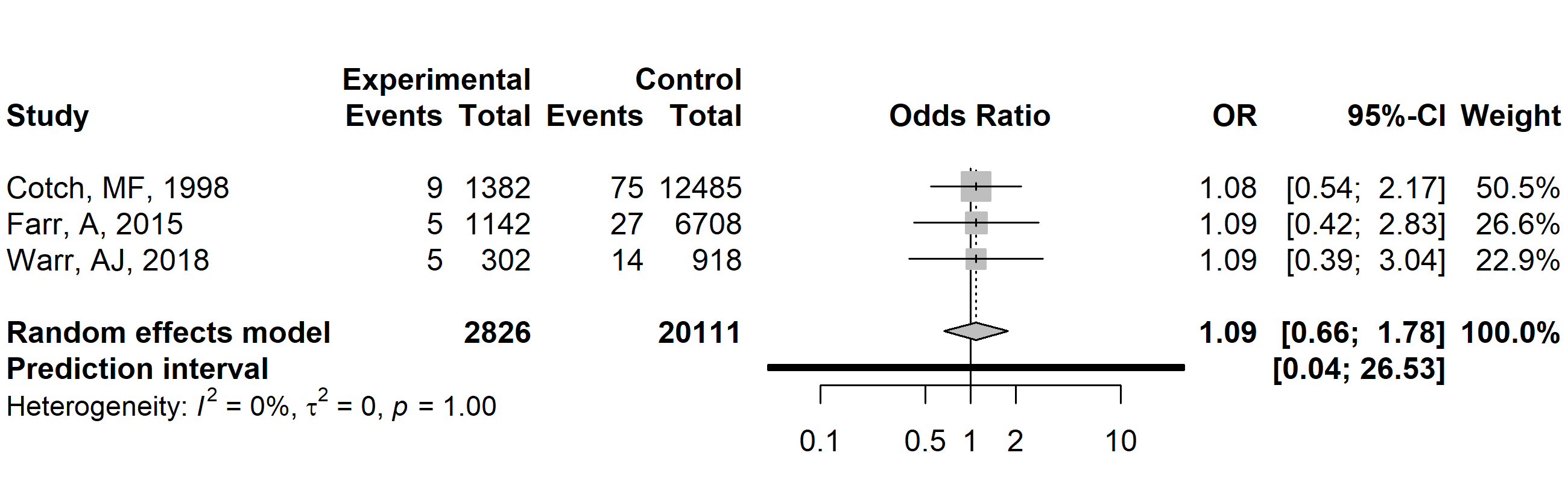


Legend: vertical line, line of no association (odds ratio 1.0); horizontal line, 95% confidence interval; vertical line inside the box, point estimate of odds ratio; grey box, study size; diamond, summary estimate with 95% confidence interval; black bar, 95% prediction interval. To the left of the line of no association, stillbirth was less likely in women with vulvovaginal yeast infection; to the right of the line of no association, stillbirth was more likely.

Preterm premature rupture of membranes


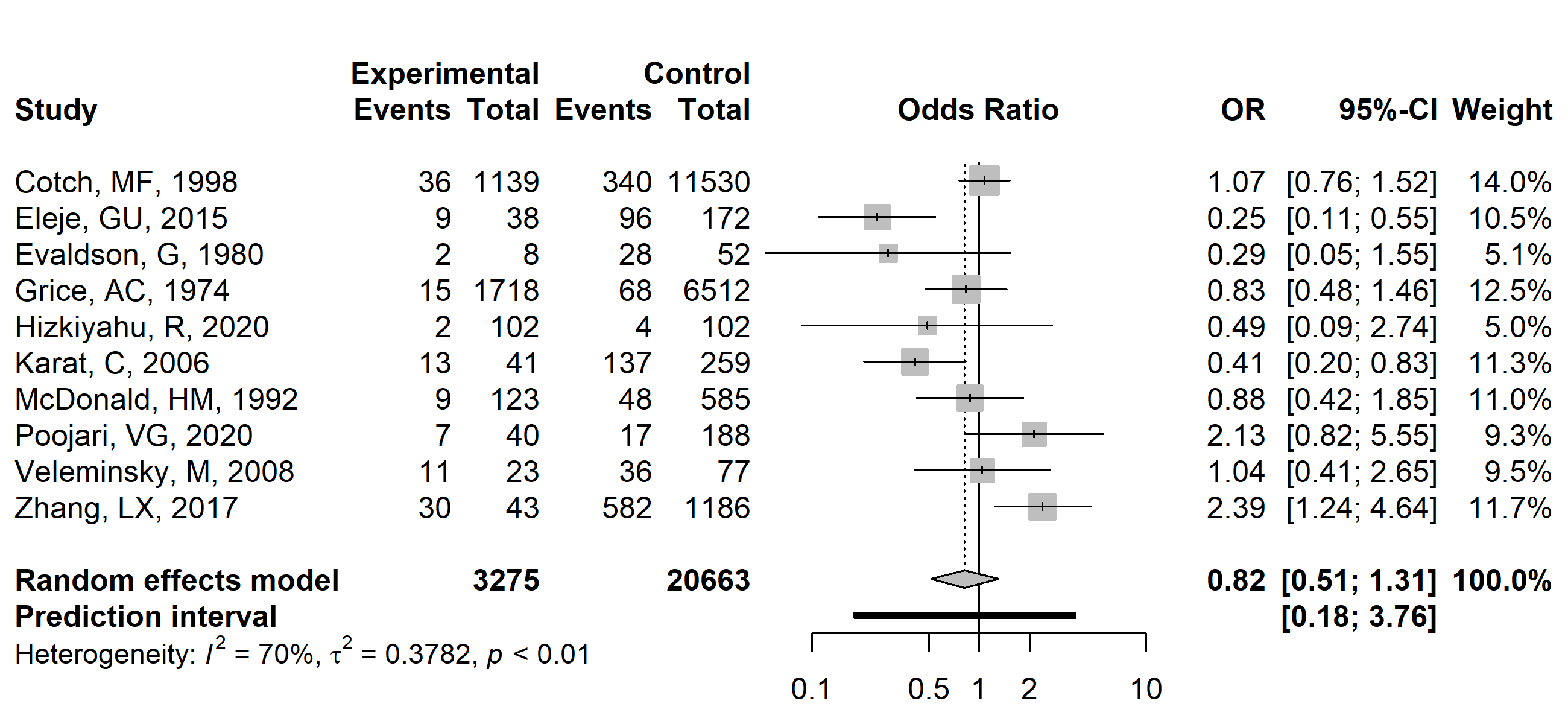


Legend: vertical line, line of no association (odds ratio 1.0); horizontal line, 95% confidence interval; vertical line inside the box, point estimate of odds ratio; grey box, study size; diamond, summary estimate with 95% confidence interval; black bar, 95% prediction interval. To the left of the line of no association, preterm premature rupture of membranes was less likely in women with vulvovaginal yeast infection; to the right of the line of no association, preterm premature rupture of membranes was more likely.

Premature rupture of membranes


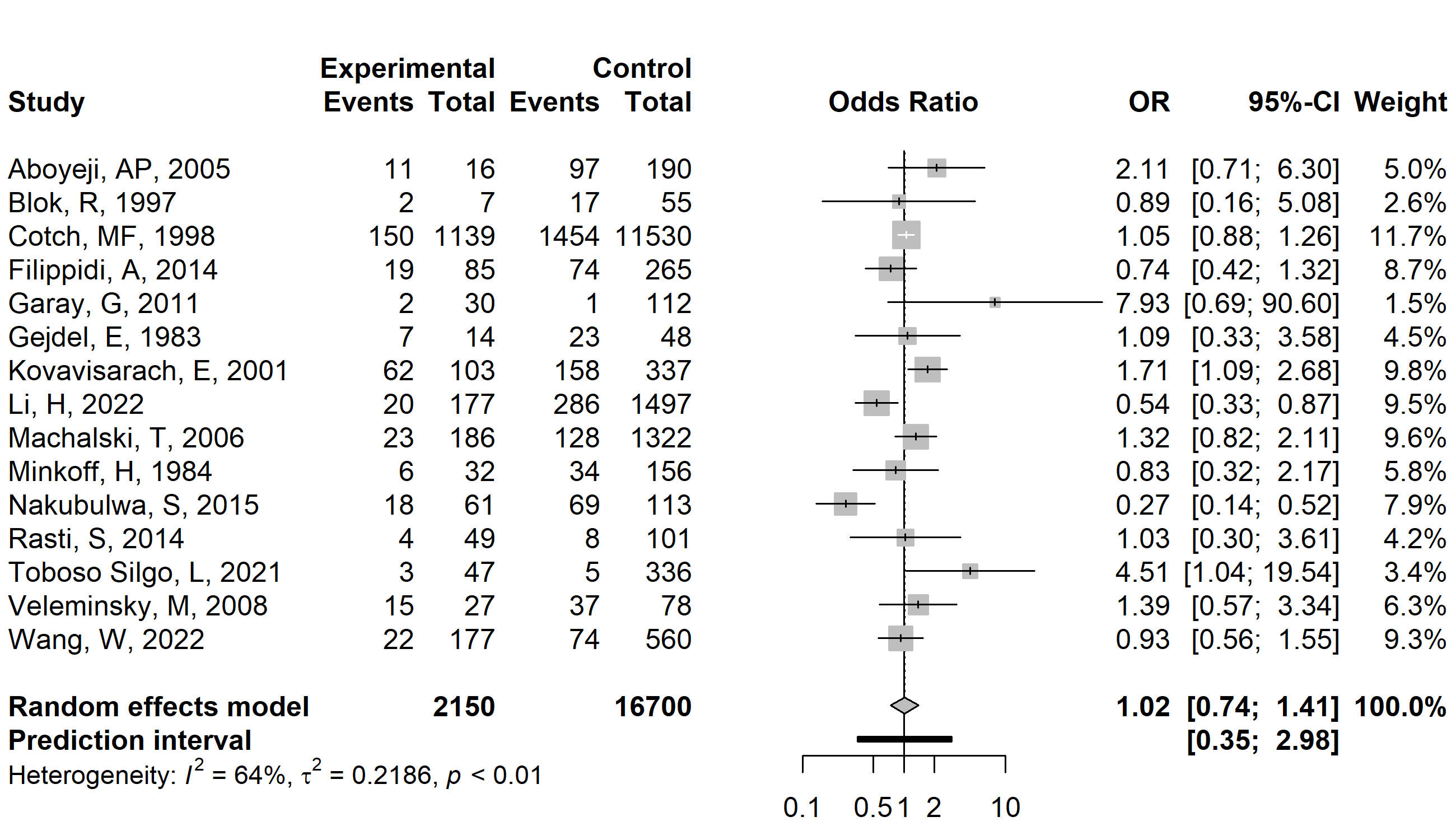


Legend: vertical line, line of no association (odds ratio 1.0); horizontal line, 95% confidence interval; vertical line inside the box, point estimate of odds ratio; grey box, study size; diamond, summary estimate with 95% confidence interval; black bar, 95% prediction interval. To the left of the line of no association, premature rupture of membranes was less likely in women with vulvovaginal yeast infection; to the right of the line of no association, premature rupture of membranes was more likely.

Low birth weight


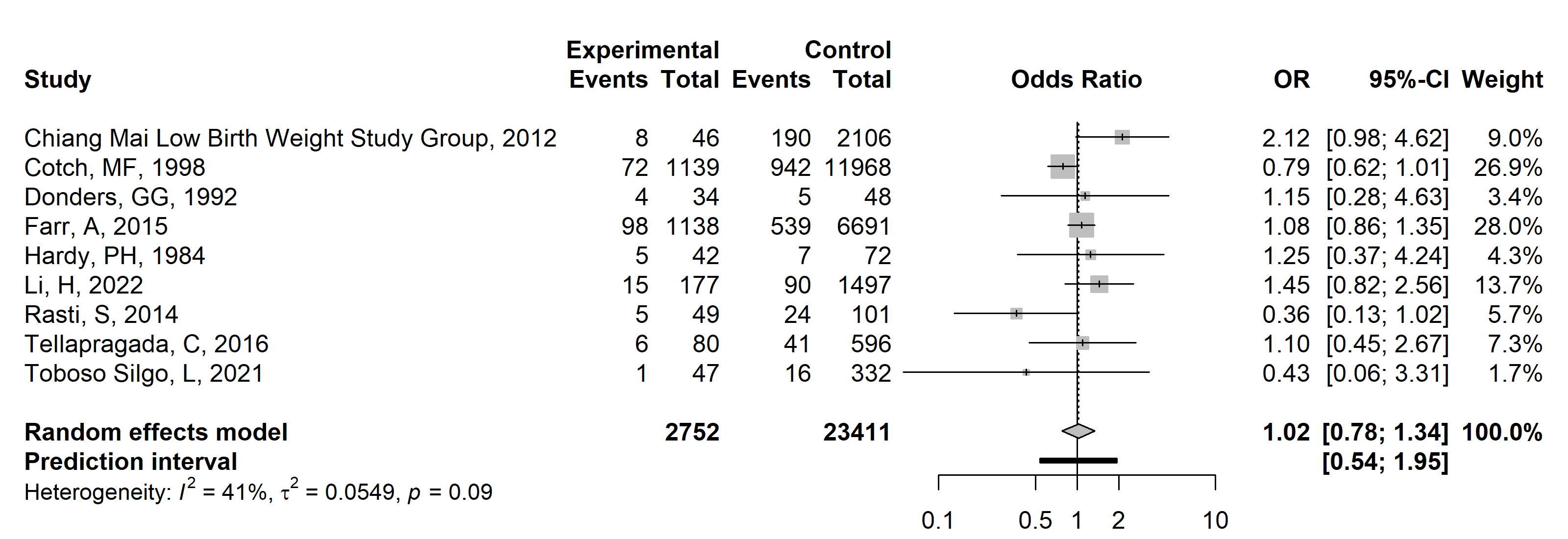


Legend: vertical line, line of no association (odds ratio 1.0); horizontal line, 95% confidence interval; vertical line inside the box, point estimate of odds ratio; grey box, study size; diamond, summary estimate with 95% confidence interval; black bar, 95% prediction interval. To the left of the line of no association, low birth weight was less likely in women with vulvovaginal yeast infection; to the right of the line of no association, low birth weight was more likely.

Inflammation of the placenta or uterus


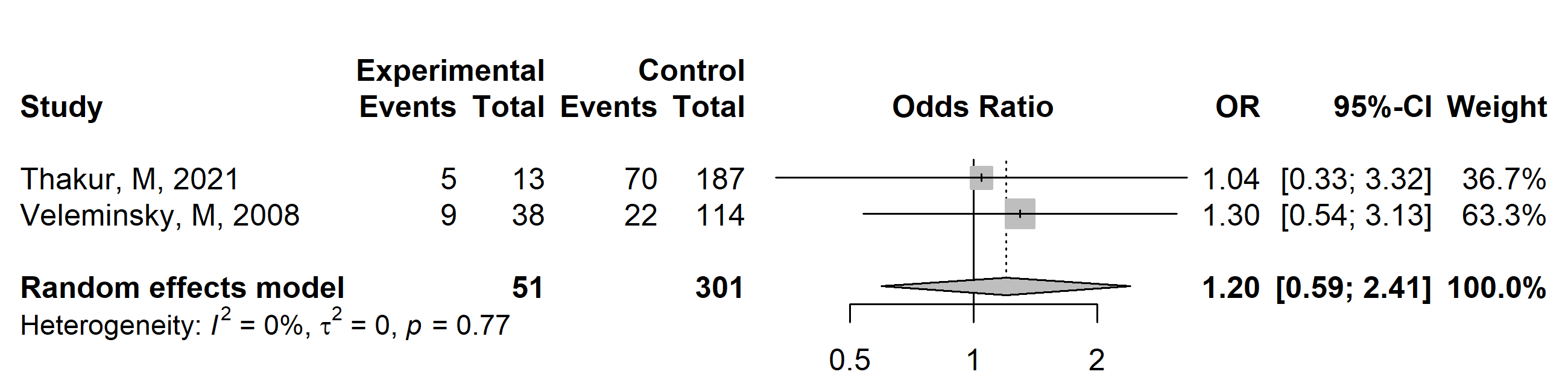


Legend: vertical line, line of no association (odds ratio 1.0); horizontal line, 95% confidence interval; vertical line inside the box, point estimate of odds ratio; grey box, study size; diamond, summary estimate with 95% confidence interval; black bar, 95% prediction interval. To the left of the line of no association, inflammation of the placenta or uterus was less likely in women with vulvovaginal yeast infection; to the right of the line of no association, inflammation of the placenta or uterus was more likely.
