# Supplementary material for: Vulvovaginal yeast infections during pregnancy and perinatal outcomes: systematic review and meta-analysis
Source: BMC Womens Health. 2023 Mar 21;23:116. doi: 10.1186/s12905-023-02258-7 (PMC10029297; doi:10.1186/s12905-023-02258-7)
Supplement: Supplementary file 5 — Additional file 5. Summary of risk of bias assessment for cohort studies, cross-sectional studies, clinical trials, and case–control studies. [file 12905_2023_2258_MOESM5_ESM.docx]

**Additional file 5 - Summary of risk of bias assessment**

Summary of risk of bias assessment for cohort studies, cross-sectional studies and clinical trials

|  | Was assessing this association an objective?* | Was selection of exposed and non-exposed cohorts drawn from the same population? | Can we be confident in the assessment of exposure? | Can we be confident that the outcome of interest was not present at start of study? | Did the statistical analysis adjust for confounding factors?* | Can we be confident in the assessment of the presence or absence of prognostic factors? | Can we be confident in the assessment of outcome? | Was the follow up of cohorts adequate? | Were co-interventions similar between groups? |
| --- | --- | --- | --- | --- | --- | --- | --- | --- | --- |
| **Cohort studies** |  |  |  |  |  |  |  |  |  |
| Blok 1997 |  |  |  |  |  |  |  |  |  |
| Chiang Mai Low Birth Weight Study Group 2012 |  |  |  |  |  |  |  |  |  |
| Cotch 1998 |  |  |  |  |  |  |  |  |  |
| Czajka 2010 |  |  |  |  |  |  |  |  |  |
| Donders 1992 |  |  |  |  |  |  |  |  |  |
| Farr 2015 |  |  |  |  |  |  |  |  |  |
| Filippidi 2014 |  |  |  |  |  |  |  |  |  |
| Frerich 1977 |  |  |  |  |  |  |  |  |  |
| Garay 2011 |  |  |  |  |  |  |  |  |  |
| **Cohort studies (continue)** | | | | | | | | | |
| Goel 2018 |  |  |  |  |  |  |  |  |  |
| Grice 1974 |  |  |  |  |  |  |  |  |  |
| Hardy 1984 |  |  |  |  |  |  |  |  |  |
| Hu 2019 |  |  |  |  |  |  |  |  |  |
| McDonald 1992 |  |  |  |  |  |  |  |  |  |
| McGregor 1990 |  |  |  |  |  |  |  |  |  |
| McLennan 1969 |  |  |  |  |  |  |  |  |  |
| Meis 1995 |  |  |  |  |  |  |  |  |  |
| Minkoff 1984 |  |  |  |  |  |  |  |  |  |
| Nadisauskiene 1995 |  |  |  |  |  |  |  |  |  |
| Payne 2016 |  |  |  |  |  |  |  |  |  |
| Payne 2021 |  |  |  |  |  |  |  |  |  |
| Polk 1989 |  |  |  |  |  |  |  |  |  |
| **Cohort studies (continue)** | | | | | | | | | |
| Poojari 2020 |  |  |  |  |  |  |  |  |  |
| Rasti 2014 |  |  |  |  |  |  |  |  |  |
| Ravindran 2021 |  |  |  |  |  |  |  |  |  |
| Rittenschober-Bohm 2018 |  |  |  |  |  |  |  |  |  |
| Schwab 2016 |  |  |  |  |  |  |  |  |  |
| Sule-Odu 2020 |  |  |  |  |  |  |  |  |  |
| Tellapragada 2016 |  |  |  |  |  |  |  |  |  |
| Tellapragada 2017 |  |  |  |  |  |  |  |  |  |
| Toboso Silgo 2021 |  |  |  |  |  |  |  |  |  |
| Usui 2002 |  |  |  |  |  |  |  |  |  |
| van Rensburg 1992 |  |  |  |  |  |  |  |  |  |
| Wang 2022 |  |  |  |  |  |  |  |  |  |
| Warr 2018 |  |  |  |  |  |  |  |  |  |
| **Cross-sectional studies** | | | | | | | | | |
| Ghaddar 2020 |  |  |  |  |  |  |  |  |  |
| Giraldo 2012 |  |  |  |  |  |  |  |  |  |
| Hizkiyahu 2020 |  |  |  |  |  |  |  |  |  |
| Li 2022 |  |  |  |  |  |  |  |  |  |
| Liu 2018 |  |  |  |  |  |  |  |  |  |
|  | | | | | | | | | |
| **Clinical trials** | | | | | | | | | |
| Kiss 2004 |  |  |  |  |  |  |  |  |  |
| Roberts 2011 |  |  |  |  |  |  |  |  |  |
| low risk of bias; higher risk of bias; high risk of bias  *Question added to the tool  The tool gives 4 answer options for each question (definitely yes, probably yes, probably no, definitely no) but only 3 risk of bias levels (low risk, higher risk and high risk), therefore we summarised the middle 2 answer options (probably yes and probably no) into one risk of bias level (higher risk). | | | | | | | | | |

Summary of risk of bias assessment for case-control studies

|  | Was assessing this association an objective?* | Can we be confident in the assessment of exposure? | Can we be confident that cases had developed the outcome of interest and controls had not? | Were the cases (those who were exposed and developed the outcome of interest) properly selected? | Were the controls (those who were exposed and did not develop the outcome of interest) properly selected? | Were cases and controls matched according to important prognostic variables or was statistical adjustment carried out for those variables? |
| --- | --- | --- | --- | --- | --- | --- |
| **Case-control studies** | | | | | | |
| Aboyeji 2005 |  |  |  |  |  |  |
| Eleje 2015 |  |  |  |  |  |  |
| Evaldson 1980 |  |  |  |  |  |  |
| Gejdel 1983 |  |  |  |  |  |  |
| Holst 1994 |  |  |  |  |  |  |
| Karat 2006 |  |  |  |  |  |  |
| Kovavisarach 2001 |  |  |  |  |  |  |
| Machalski 2006 |  |  |  |  |  |  |
| Maimoona 2001 |  |  |  |  |  |  |
| Nakubulwa 2015 |  |  |  |  |  |  |
| Sandeva 2016 |  |  |  |  |  |  |
| Thakur 2021 |  |  |  |  |  |  |
| **Case-control studies (continue)** | | | | | | |
| Thomas 2015 |  |  |  |  |  |  |
| Veleminsky 2008 |  |  |  |  |  |  |
| Zhang 2017 |  |  |  |  |  |  |
| low risk of bias; higher risk of bias; high risk of bias  *Question added to the tool  The tool gives 4 answer options for each question (definitely yes probably yes probably no definitely no) but only 3 risk of bias levels (low risk higher risk and high risk) therefore we summarised the middle 2 answer options (probably yes and probably no) into one risk of bias level (higher risk). | | | | | | |
