# Supplementary material for: Vulvovaginal yeast infections during pregnancy and perinatal outcomes: systematic review and meta-analysis
Source: BMC Womens Health. 2023 Mar 21;23:116. doi: 10.1186/s12905-023-02258-7 (PMC10029297; doi:10.1186/s12905-023-02258-7)
Supplement: Supplementary file 6 — Additional file 6. Funnel plots of secondary outcomes preterm premature rupture of membranes and premature rupture of membranes. [file 12905_2023_2258_MOESM6_ESM.docx]

**Additional file 6 - Funnel plots of secondary outcomes**

Preterm premature rupture of membranes


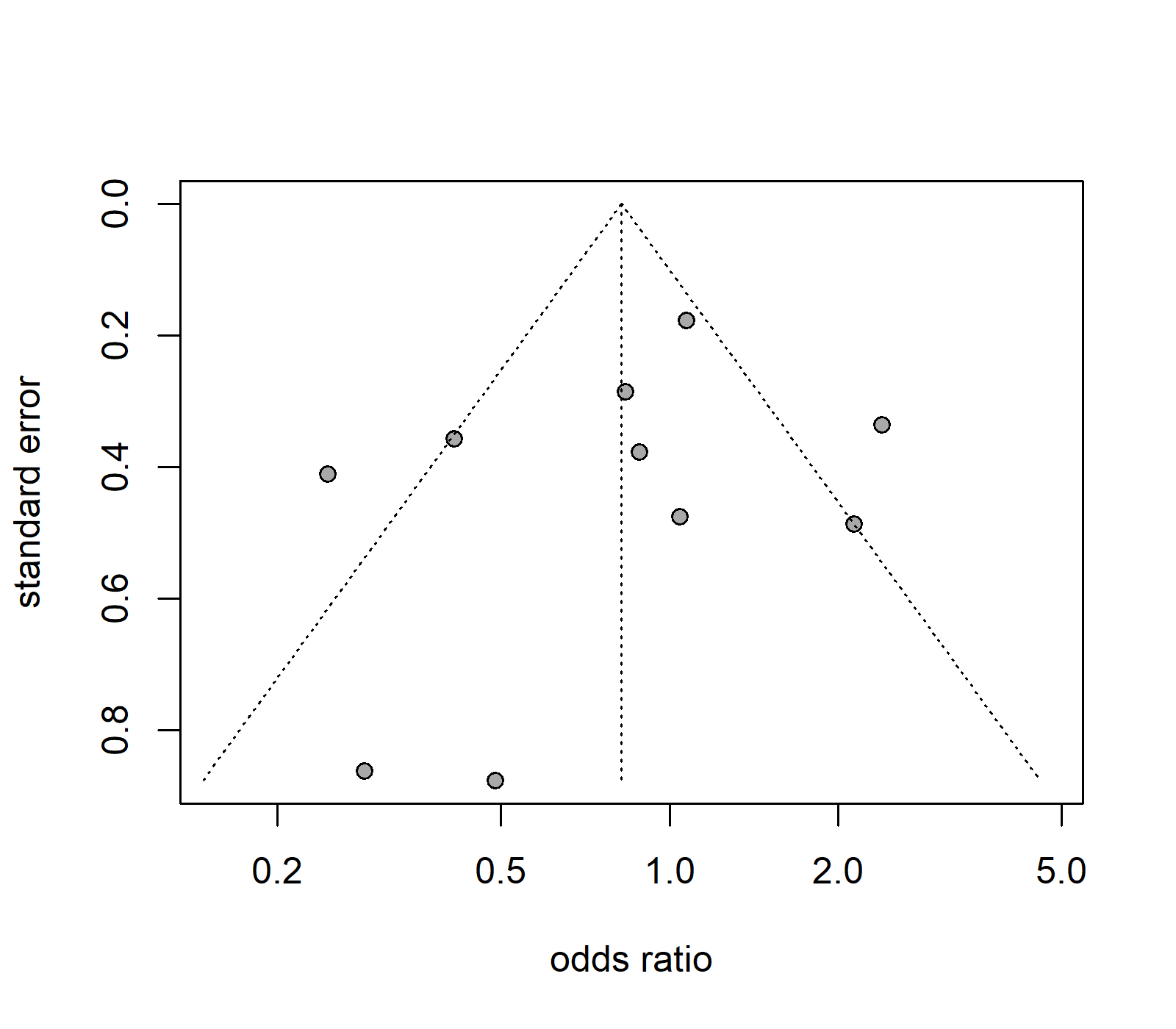


Legend: circle, represents one study; triangle, region where 95% of the data points would lie in absence of small study biases; vertical dashed line, odds ratio from meta-analysis

Premature rupture of membranes


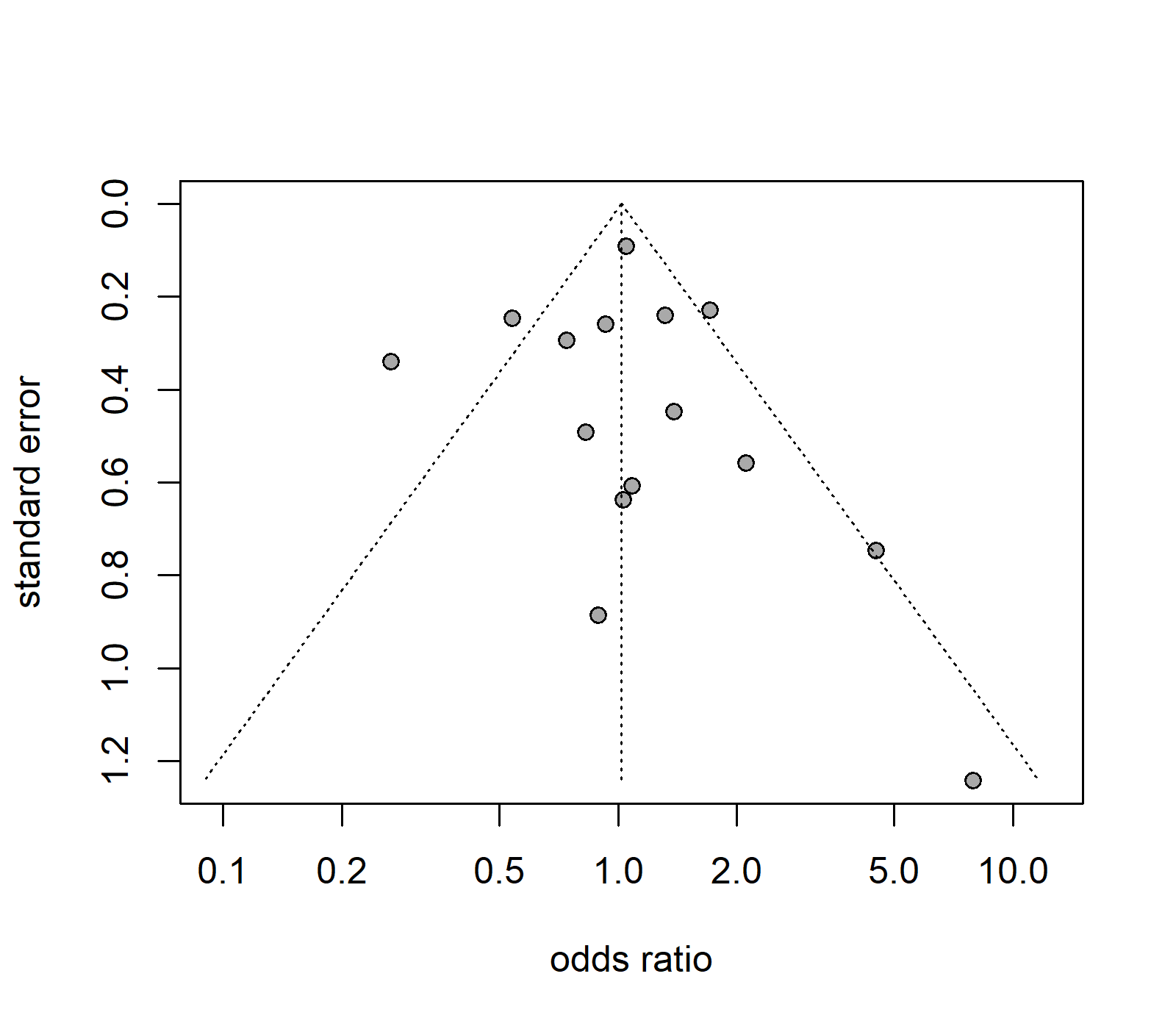


Legend: circle, represents one study; triangle, region where 95% of the data points would lie in absence of small study biases; vertical dashed line, odds ratio from meta-analysis
